# Detecting cryptic clinically relevant structural variation in exome-sequencing data increases diagnostic yield for developmental disorders

**DOI:** 10.1016/j.ajhg.2021.09.010

**Published:** 2021-10-08

**Authors:** Eugene J. Gardner, Alejandro Sifrim, Sarah J. Lindsay, Elena Prigmore, Diana Rajan, Petr Danecek, Giuseppe Gallone, Ruth Y. Eberhardt, Hilary C. Martin, Caroline F. Wright, David R. FitzPatrick, Helen V. Firth, Matthew E. Hurles

**Affiliations:** 1 Wellcome Sanger Institute, Wellcome Genome Campus, Cambridge, Hinxton CB10 1SA, UK; 2 Department of Human Genetics, KU Leuven, Herestraat 49, Box 602, Leuven 3000, Belgium; 3 University of Exeter Medical School, Institute of Biomedical and Clinical Science, Royal Devon and Exeter Hospital, Exeter EX2 5DW, UK; 4 MRC Human Genetics Unit, Institute of Genetics and Cancer, University of Edinburgh, WGH, Edinburgh EH4 2SP, UK; 5 East Anglian Medical Genetics Service, Box 134, Cambridge University Hospitals NHS Foundation Trust, Cambridge Biomedical Campus, Cambridge CB2 0QQ, UK

**Keywords:** structural variation, insertions/deletions, developmental disorders, bioinformatics, diagnostics

## Abstract

Structural variation (SV) describes a broad class of genetic variation greater than 50 bp in size. SVs can cause a wide range of genetic diseases and are prevalent in rare developmental disorders (DDs). Individuals presenting with DDs are often referred for diagnostic testing with chromosomal microarrays (CMAs) to identify large copy-number variants (CNVs) and/or with single-gene, gene-panel, or exome sequencing (ES) to identify single-nucleotide variants, small insertions/deletions, and CNVs. However, individuals with pathogenic SVs undetectable by conventional analysis often remain undiagnosed. Consequently, we have developed the tool InDelible, which interrogates short-read sequencing data for split-read clusters characteristic of SV breakpoints. We applied InDelible to 13,438 probands with severe DDs recruited as part of the Deciphering Developmental Disorders (DDD) study and discovered 63 rare, damaging variants in genes previously associated with DDs missed by standard SNV, indel, or CNV discovery approaches. Clinical review of these 63 variants determined that about half (30/63) were plausibly pathogenic. InDelible was particularly effective at ascertaining variants between 21 and 500 bp in size and increased the total number of potentially pathogenic variants identified by DDD in this size range by 42.9%. Of particular interest were seven confirmed *de novo* variants in *MECP2*, which represent 35.0% of all *de novo* protein-truncating variants in *MECP2* among DDD study participants. InDelible provides a framework for the discovery of pathogenic SVs that are most likely missed by standard analytical workflows and has the potential to improve the diagnostic yield of ES across a broad range of genetic diseases.

## Main text

Structural variation (SV) includes a diverse collection of genomic rearrangements such as copy number variation (CNV), mobile element insertions (MEIs), inversions, translocations, and others.^1^ Depending on population ancestry and technology used, the typical human genome harbors between 7,000 and 25,000 polymorphic SVs, with the majority constituting bi-allelic CNVs and MEIs.^2^ While most SVs have minimal, if any, functional impact, SVs have been recognized as causative variants in congenital disorders.^3^, ^4^, ^5^

In diagnostic testing of suspected genetic disorders, SVs are often identified using chromosomal microarrays (CMAs) which offer a low-cost albeit low-resolution method for the identification of large CNVs (typically >20 kbp in length for genic regions). CMAs are still widely used by diagnostic laboratories despite the increasing maturity of genome sequencing-based tools for SV discovery^6^ and the wealth of clinically ascertained exome-sequencing (ES) data already generated for the ascertainment of single-nucleotide variants (SNVs) and small insertions/deletions (indels).^7^ There are several reasons for this. First, the cost, computational power, and informatics complexity necessary for genome sequencing-based diagnostics is still a barrier to many public and private healthcare providers.^8^ Second, current ES-based SV-discovery approaches focus on methods that interrogate sequencing coverage to identify regions of copy number variation within one genome compared to others.^9^ As such, ascertainment is typically limited to CNVs of size >10 kbp, with resolution largely a factor of the sequencing depth and the density and number of baits in the ES assay, analogous to probes in CMAs. Thus, despite potentially offering improvements in CNV ascertainment over CMAs, ES as a tool for the assessment of diagnostic SVs has been slow to enter the clinic.^10^

Consequently, individuals with genetic abnormalities smaller than the discovery resolution of CMA or standard SV-ES approaches (>10 kbp) but larger than variants able to be accurately called using typical SNV/indel-based approaches (<50 bp)^11^ often remain undetected, here termed “cryptic.” To address this unmet need, we have developed the tool InDelible, which examines ES data for split read pairs indicative of SV breakpoints. We decided to focus on split reads because the formation of unique junction sequences is a shared characteristic of a broad range of different classes of SVs. We applied InDelible to ES data generated from 13,438 probands with severe developmental disorders (DDs) recruited as part of the Deciphering Developmental Disorders (DDD) study. Approximately 29% of DDD probands harbor a pathogenic *de novo* mutation in a gene known to be associated with DD^7^ and have been previously assessed for a wide range of variant classes such as coding,^7^ noncoding,^12^ and splice site^13^ SNVs and indels, multinucleotide variants,^14^ mobile element insertions,^3^ and copy number variants (unpublished data). As such, the DDD study represents an ideal opportunity to demonstrate the additive diagnostic potential of identification of SVs at scale using split-read information.

**Figure 1:**
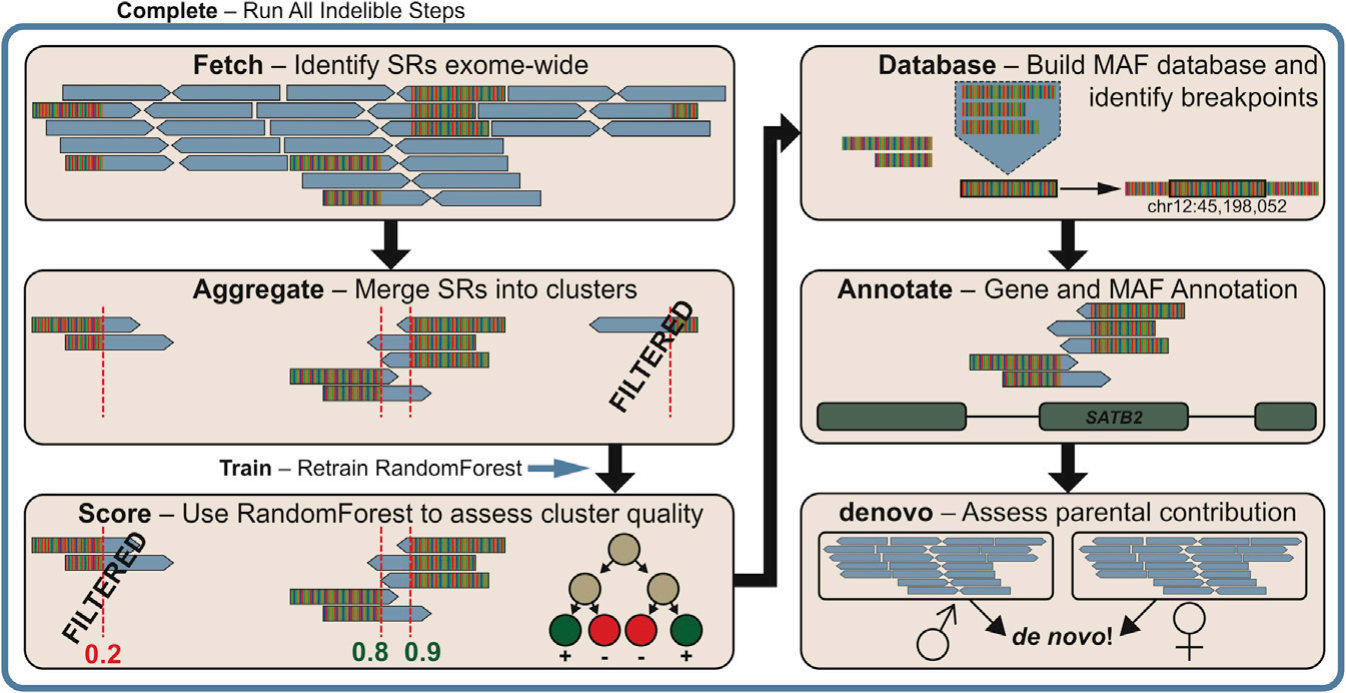
InDelible SV discovery in ES data InDelible processes one ES sample provided in BAM or CRAM format via six primary steps (tan boxes). First, alignment files are queried for all reads where part of the aligned sequence matches the reference genome and the other does not (i.e., split reads; Fetch). Next, reads are clustered (Aggregate) and scored using a random forest model^15^ trained using a variant truth set (Score; see Figure S1 and supplemental material and methods for more detail). Split reads are then merged within clusters across individuals to determine the longest quality junction sequence and mapped back to the genome with bwa mem^16^ and to a set of curated repeats with blastn.^17^ These alignments are then used to determine breakpoint frequency, likely breakpoints, length, and structural variant class (i.e., deletion, duplication, insertion, etc.; Database). Split read clusters are subsequently annotated with population frequency and intersection with genomic functional annotations, such as protein-coding genes (Annotate). Finally, clusters are assessed for presence or absence in parental samples, where available, to determine inheritance status and identify likely *de novo* variants (denovo). All of these commands can be run on one sample via the “Complete” command (blue box). InDelible also includes the “Train” command to train a new random forest model from user-provided labeled training data.

InDelible variant discovery and analysis proceeds in several steps (Figure 1; detailed description in supplemental material and methods). In summary, InDelible identifies split reads, aggregates them into clusters at the same genomic location, filters these clusters to remove technical artifacts and retain likely genetic variants, and then combines unaligned portions of split reads and maps them to the genome to characterize the nature of the variant. InDelible also calculates the frequency of each split-read cluster across a population of individuals to facilitate the filtering of variants on the basis of minor allele frequency.

InDelible is coded in Python, uses the pysam (see web resources) library for sequence alignment file manipulation (Table S1), and works on bwa-aligned BAM or CRAM format files.^18^ We have designed InDelible to be scalable for datasets comprising individual probands to multi-thousand sample cohorts and our estimates suggest that, to analyze a dataset of 1,000 trios, InDelible would require approximately 1556 CPU h, or 15.6 h of real time on a 100-core compute cluster (Figure S2). Additionally, for easy implementation on cloud compute platforms, we have made InDelible available as a Docker image (see supplemental material and methods and data and code availability).

We benchmarked InDelible against GATK^11^ and Manta,^19^ another SV detector which utilizes split reads, for variants across a range of allele frequencies and sizes. First, we ran these callers on ES data generated for a control individual by the Genome in a Bottle Consortium.^20^^,^^21^ We then used the gold-standard variant dataset provided by the Genome in a Bottle Consortium for the same individual, which amalgamates variant-call data across several data types including whole genome short-, linked-, and long-read sequencing, to assess recall and specificity of the resulting ES calls for each algorithm (Figure S3; supplemental material and methods). When using ES data, InDelible equals or exceeds the recall of both GATK and Manta for variants between 21 and 10 kbp in length, the variant space InDelible was targeted to identify. Relative to InDelible, GATK and Manta had 81.7% and 15.0% recall for deletions >20 bp in length, respectively, and 86.9% and 8.2% recall for insertions >20 bp in length, respectively. In this same experiment InDelible has moderately increased false discovery rates compared to GATK (Figure S3). These issues can likely be attributed to InDelible being designed for maximum sensitivity in clinical sequencing data and can likely be abrogated via the design of better hard filters when analyzing population-level variants and/or retraining the random forest using training data from population-level datasets.

A key objective for the design of InDelible was to identify *de novo* variants potentially causative of a proband’s disorder. As such, variants are primarily filtered on: (1) the population frequency of the split read cluster to remove variants too common to be plausibly causative of a rare disorder, (2) absence in unaffected parents (when available), and (3) intersection of variant breakpoints with the coding sequences of known-disease-associated genes. Defining the precise molecular structure of SVs from short read sequencing data can be challenging, and even minor errors in breakpoint precision can have large consequences on interpretation (e.g., in- versus out-of-frame indels). Hence, we opted to identify all variants which intersect relevant DD-associated genes for further manual curation rather than relying on generic variant interpretation tools.

To evaluate the utility of InDelible for diagnostic analyses, we applied InDelible to identify putatively diagnostic variants in 13,438 probands recruited to the DDD study. Probands were exome sequenced either with both parents (trios, n = 9,848) or with one or both parents absent (non-trios, n = 3,590). We first identified split reads and split read clusters (Figure 1) to ascertain 353,313,108 redundant split read clusters across all probands. Random forest filtering resulted in retention of 30,667,420 high-quality, redundant split read clusters across all probands, or 8.7% of originally ascertained loci (supplemental material and methods, Figure S4). After cluster filtering, we merged all retained clusters into a set of 1,954,642 non-redundant split read clusters across all 13,438 probands, with 1,342,050 (68.7%) clusters found only in one proband (Figure S5). Clusters were evenly distributed across all chromosomes as a function of chromosome length (r^2^ = 0.739; Figure S6). Retained clusters were then annotated with putative breakpoints, intersecting gene(s), and population frequency. InDelible was also able to determine the missing 5′ or 3′ breakpoint, variant length, and variant type (i.e., deletion, duplication, MEI, etc.) of 199,932 (10.2%) clusters (supplemental material and methods). Of the clusters which InDelible was able to resolve to a specific variant type, 65.7% were simple deletions/duplications, with the remainder comprising complex events, MEIs, translocations/segmental duplications, and non-templated insertions (Figure S7). Ascertainment of variant type and length are dependent on sequencing depth and population frequency (Figure S5) but are optimized for the length of variants InDelible is best suited to identify (∼20–500 bp; Figure S8). This specificity is best demonstrated when restricting to clusters that are plausibly associated with DDD study participant phenotype (see below); InDelible accurately resolves both breakpoints, length, and variant type for 86.3% (126/146) of such clusters (Table S2).

**Figure 2:**
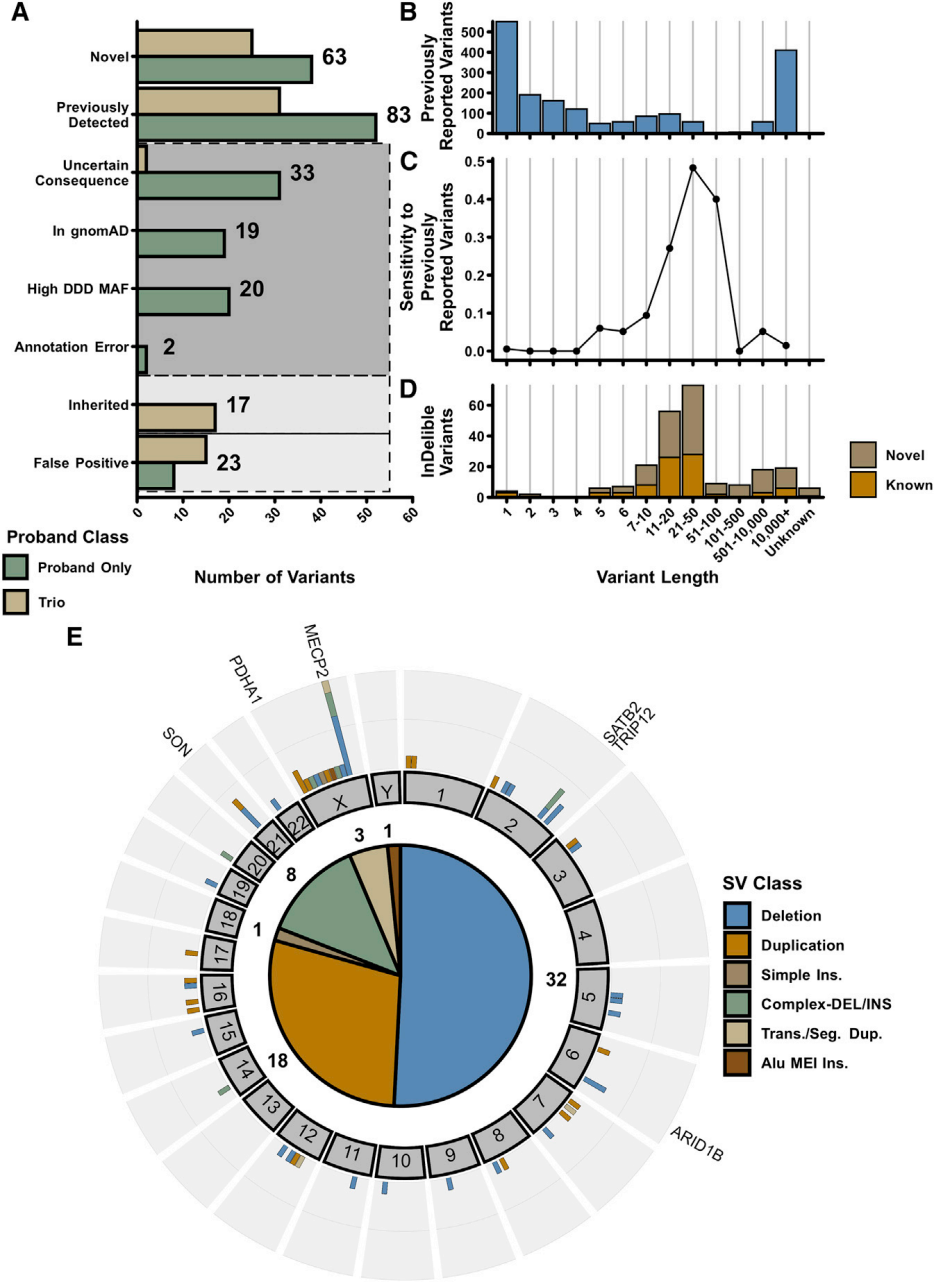
SV ascertainment in the DDD study with InDelible (A) Breakdown of putative variant consequences for all 260 variants identified in this study delineated by whether or not the proband was sequenced with both parents (trio, tan) or not (proband only, dark green). Light gray and dark gray boxes represent erroneous variants and variants unlikely to be associated with a proband phenotype, respectively. (B) Total number of DDD variants reported to referring clinicians via the DECIPHER platform among DDD probands with a net size change ≥1 bp. (C) Sensitivity of InDelible to DDD variants reported to referring clinicians via the DECIPHER platform among various variant size bins. (D) Categorization of InDelible-ascertained variants into previously known (orange) versus those novel (brown) to this study based on size. (E) Distribution of variants unique to InDelible throughout the genome. Shown in the outer plot are the total number of InDelible variants per gene, with genes that have multiple previously undetected variants labeled. Displayed in the inner plot are the total number of variants for each SV type identified.

We next restricted our variant set to rare (call frequency < 0.04%) clusters found only in or near (here defined as within ±10 bp of any exon) the coding sequence of 399 dominant or X-linked DD-associated genes from the Developmental Disorders Genotype-to-Phenotype database (DDG2P).^22^ Variants identified within individuals sequenced as a parent-offspring trio were then also assessed for *de novo* status. Filtering on allele frequency, inheritance, and gene intersection resulted in a preliminary set of 260 candidate indels and SVs across all 13,438 probands (Figure 2A; Table S2; supplemental material and methods). Based on manual variant inspection,^23^ we determined that 2/260 (0.8%) were erroneously annotated to have intersected a mono-allelic DD gene, 17/260 (6.5%) candidate *de novo* events were likely to be present in a parent (i.e., parental false negatives), and 23/260 (8.8%) were unlikely to be real variants (i.e., offspring false positives). Four probands contributed 52.2% of false positive variants, indicating that sample selection and/or additional sample-level QC could further lower the false positive rate of InDelible (Figure 2A; Table S2).

Following variant quality control, we further curated variants for those likely to be associated with a proband phenotype (Figure 2A). We considered variants with a non-Finnish European minor allele frequency of ≥1 × 10^−4^ (19/260; 7.3%) in the Genome Aggregation Database (gnomAD)^1^^,^^24^ or presence in other unrelated individuals within DDD (20/260; 7.7%) as unlikely to be the cause of the child’s disorder. Additionally, variants confined to introns or 5′/3′ UTRs were also defined as variants of uncertain significance and were not considered further (33/260; 12.7%). This final round of filtering left 146 SVs and large indels which could plausibly explain a proband phenotype (56 from probands sequenced as trios, 90 from non-trio probands).

We next sought to determine the sensitivity of InDelible to clinically relevant variants ascertained using alternative methods. DDD has already identified (across both trio and non-trio probands) 1,853 rare, plausibly pathogenic variants with a net size difference ≥1 bp (i.e., non-SNVs) in the same DDG2P gene set defined above^7^—variants potentially detectable with a split read-based method such as that employed by InDelible. The majority of these variants are private or low-allele frequency small indels between 1 and 10 bp in size (1,218/1,853; 65.7%) or large CMA or ES-ascertained CNVs ≥10 kbp in length (410/1,853; 22.1%; Figure 2B). As anticipated due to the low number of split reads at variant breakpoints as variant size decreases, InDelible performed poorly in identification of very short variants ≤10 bp with an overall sensitivity of 1.4% (Figure 2C). Sensitivity improved as a function of variant size, peaking at 48.3% sensitivity for variants between 21 and 50 bp, but dropped again for variants ≥100 bp. To better understand why InDelible missed such variants, we manually curated the 34 potentially pathogenic variants between 21 and 500 bp not identified by InDelible. We found that InDelible missed variants for three primary reasons. First, these potentially pathogenic variants include some higher-frequency variants that are too common to be plausibly pathogenic whose true allele frequency was underestimated previously, but have now been more accurately determined by InDelible and thus subsequently filtered out (n = 12/34; 35.3%). Second, several variants have low split read support (i.e., <5 reads) despite being located in high-coverage regions and were thus excluded by our stringent filtering approach (n = 11/34; 32.4%). Third, as variant size increases, it becomes more likely that the breakpoints of SVs which impact coding sequence lie outside of ES target regions (i.e., within intronic and intergenic sequences). Ergo, such variants are refractory to identification with split reads and likely to be missed by any split-read caller (n = 6/34; 17.6%). Combined, these three explanations account for 85.3% of variants between 21 and 500 bp missed by InDelible. While variants with breakpoints outside of sequencing baits are invisible to InDelible, additional fine-tuning of InDelible’s filtering parameters could, in theory, output variants with lower split read support or variants with higher allele frequencies.

These 63 previously undetected variants (four of which were ascertained by an earlier version of InDelible and included as part of a previous DDD publication^25^) that impact known DD-associated genes (Table S2) are composed primarily of deletions and duplications (50/63; 79.4%) but also includes variants with diverse mutational mechanisms such as MEIs, complex rearrangements, and dispersed duplications/translocations (Figure 2E). 25 of these variants were observed in trio probands, with parental data supporting a *de novo* origin for all of these variants. InDelible was particularly effective at identifying variants between 21 and 500 bp in size (Figure 2D); 30 previously undetected variants (47.6% of InDelible-specific variants) lie within this size range and represent a 42.9% increase in putatively pathogenic variants 21–500 bp in length among DDD probands (Figure 2D). We also identified six genes with multiple previously undetected SVs among unrelated individuals, of which the most recurrently affected was *MECP2,* the causal gene of Rett syndrome (Figure 2E).^26^

From an initial round of clinical review, based on intersecting gene(s) and associated phenotypes, we concluded that nine (14.3%) of these 63 previously undetected variants were unlikely to explain the referred proband’s phenotype, and were thus excluded from future analysis (Table S2). We next attempted PCR validation of the 54 putatively pathogenic variants (supplemental material and methods). Of the variants for which conclusive validation results could be obtained, 23/23 (100%) were confirmed as true positives, either by the obvious presence of a mutant band of expected size with gel electrophoresis or by follow-up capillary sequencing where the gel result was uncertain (Table S2). For variants for which PCR was possible, we also confirmed that 10/10 (100%) putative *de novo* variants identified in trio probands were indeed absent from both parents.

All 54 plausible pathogenic variants were reported to referring clinicians and clinically interpreted by two senior clinical geneticists; 30/54 (55.6%) were classified as pathogenic or likely pathogenic by both clinical geneticists (Table S2). Of these variants, those identified in non-trio probands (n = 31/54 plausibly pathogenic variants) for which inheritance status is unavailable, were less likely to be interpreted as being pathogenic (Fisher’s p = 0.006). This finding is corroborated by the difference in the proportion of in-frame versus out-of-frame deletions and duplications ≤50 bp between trio and non-trio probands; 80.0% of deletions and duplications are in-frame for non-trios versus 19.0% for trios (Fisher’s p = 1.5 × 10^−6^; Figure S9). This is consistent with population-level observations: out-of-frame deletions and duplications are typically under stronger negative selection than in-frame variants^27^ and an increased proportion of in-frame variants in non-trio probands is suggestive of a greater proportion being benign. The difference is likely attributable to the absence of parental data leading to the inclusion of rare benign inherited variants that are unlikely to be filtered out using population variation data (e.g., gnomAD^1^^,^^24^). Overall, *de novo* variants identified by InDelible represent 0.7% (18/2592) of all confirmed diagnoses among trio probands in the DDD study.

**Figure 3:**
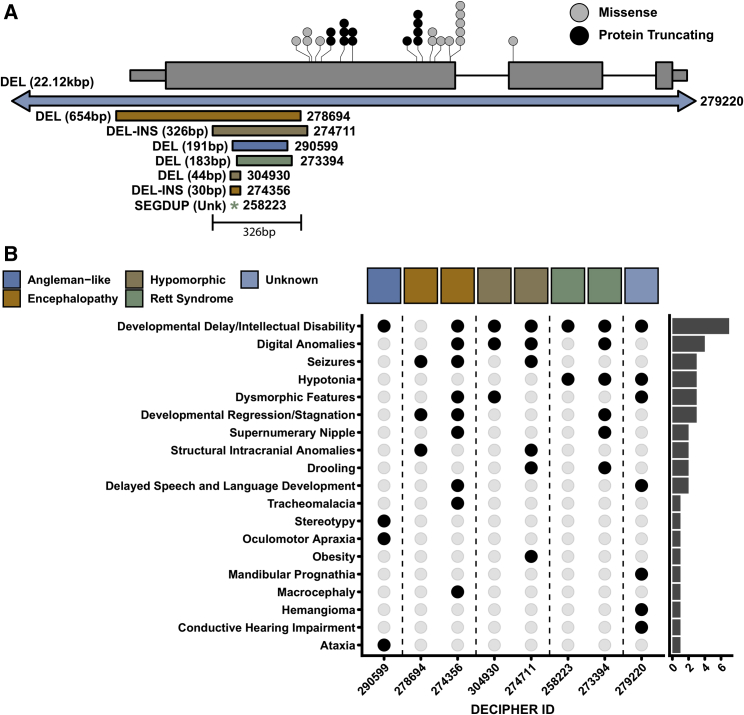
Clustered SVs in MECP2 cause diverse phenotypes (A) Shown is a cartoon representation of the gene *MECP2*, with stop-gained (black circles) and missense (gray circles) *de novo* SNVs identified in DDD trios. Each circle represents one proband, with recurrent variants represented by stacks of circles. Below the *MECP2* gene model, we have shown the seven variants identified by InDelible as well as the single whole gene deletion previously identified via CMA (proband 279220; arrows indicate this variant extends beyond the scale shown in the diagram). Sizes adjacent to variants represent the difference in number of reference and alternate bases in the indicated DDD study participant genome. We have indicated that the variant in DDD study participant 258223 only incorporates non-references bases (i.e., an insertion) with an asterisk. Variants are colored by their classification in (B). All InDelible-ascertained variants overlap the same 326 bp region in the last exon of *MECP2*. (B) Diverse proband phenotypes among *MECP2* SV carriers. Each proband carrying a *MECP2* SV from (A) is shown on the x axis, with phenotypes annotated by the referring clinician shown on the y axis. Filled black circles represent when a corresponding proband displays the corresponding phenotype. Colored boxes on the top of the plot represent the diverse phenotypes we identified following clinical review. The y axis marginal histogram represents the number of times the corresponding phenotype was observed among our SV probands.

InDelible identified a total of seven confirmed *de novo* variants ≥20 bp in length affecting *MECP2* (Figures 2E and 3A), all predicted to be protein truncating. As expected and in accordance with known sex bias among individuals with Rett syndrome,^28^ all variants were ascertained from female probands. Out of these seven probands, two have phenotypes that could be described as consistent with typical Rett syndrome presentation.^28^ Through in-depth clinical curation of HPO terms (see supplemental material and methods), we grouped probands with putative loss-of-function mutations caused by SVs in *MECP2* into four categories (Figure 3B). Cases identified by InDelible thus represent the wide variety of diverse clinical presentations that can result from disruption of the C terminus of *MECP2*^29^ and include previously observed *MECP2*-associated phenotypes such as early-onset seizures and Angelman-like symptoms (Table S3; Figure 3B).^30^

Interestingly, all five of our *MECP2* variants in probands without typical Rett syndrome presentation overlapped the same 326 bp region located within the final coding exon and, aside from a previously ascertained whole gene deletion (proband 279220), do not overlap with putatively pathogenic SNVs identified within the DDD study (Figure 3A). The SV-specific region corresponds to an area of low sequence complexity and has been previously ascertained as hyper-mutable by several studies.^29^^,^^31^ The molecular function of this region of *MECP2* is poorly understood and it is uncertain as to the consequences that our described variants may have on protein structure beyond decreasing transcript abundance and/or overall protein stability.^29^

The seven *de novo MECP2* variants constitute 28.0% (7/25) of all novel *de novo* variants identified by InDelible and 35.0% (7/20) of all confirmed *de novo* protein-truncating or gene-deleting variants of *MECP2* in the DDD study^7^ (Figure 3A).

As several publications have shown that rare, inherited variants are also important in the genetic architecture of developmental disorders,^32^ we next sought to examine whether InDelible could be used to identify such variants. We repeated our filtering as described above but limited to variants found in only a single proband with split read support from either parent (supplemental material and methods). This approach identified a total of 145 variants within the coding sequence of mono-allelic DD genes. As expected based on our analysis of variants in probands sequenced without their parents (Figure S9), a large proportion of inherited variants we identified were balanced/in-frame deletions or duplications with uncertain effect on the target protein (50; 34.5%). Others either primarily overlapped noncoding sequence, were found in an individual with a more likely diagnostic variant, were large duplications which only partially overlapped the gene of interest, were already identified based on an alternate breakpoint as part of our *de novo* analysis, or were also identified in control individuals at high enough allele frequencies to be considered unlikely to be associated with an individual’s phenotype.^1^^,^^24^ Initial filtering based on these criteria left a remainder of 17 variants for clinical interpretation.

Of the remaining inherited variants, seven were already identified via other approaches and reported to referring clinicians with six considered as likely benign and one as likely pathogenic. The remaining ten variants were referred to the same two senior clinician geneticists as for our *de novo* analysis detailed above (Table S4). Of these ten variants, all but one were unlikely to be involved in individual phenotype. The sole remaining inherited variant, an out-of-frame deletion in *KAT6B*, was identified in a proband-mother pair and was deemed a variant of uncertain consequence upon initial clinical review. Follow-up with the referring clinician regarding the mother’s phenotype determined that the mother did not exhibit any features of the proband’s disorder. As such, this variant was deemed to be likely benign. Combined, these data show that InDelible is effective at identifying rare, inherited variants but that the overall diagnostic yield may be low.

Here we present the development and application of InDelible, a tool designed for the rapid assessment of ES data for breakpoints of rare, pathogenic cryptic SVs involved in single-gene disorders (Figure 1). We applied InDelible to 13,438 proband genomes sequenced as part of the DDD study and identified a total of 146 candidate pathogenic variants impacting genes associated with dominant or X-linked DD (Figure 2A, Table S2). Of these 146 variants, 63 were not previously identified in DDD probands, despite the wide range of SV and InDel detection algorithms that have previously been deployed on this cohort.^7^^,^^25^ Notably, we increased the number of putatively diagnostic variants among DDD probands 21–500 bp in length by 42.9% (Figure 2D). Through conservative clinical assessment of these 63 variants, we determined that 30 (47.6%) of our previously undetected variants were considered likely causative of proband phenotype—of particular interest was the large number of protein-truncating SVs we identified in *MECP2* (Figure 3).

The variant size range which InDelible interrogates is complementary to other approaches commonly used for variant discovery from ES data.^9^^,^^11^ While other previously described algorithms have also attempted to mine split read information for structural variant detection,^11^^,^^19^^,^^33^ they have different properties that preclude meaningful comparison with InDelible.^11^ Some have been trained primarily on genome sequencing data rather than ES data,^19^^,^^33^ others do not explicitly assess *de novo* status, and many are not readily scalable to a dataset of ∼10,000 trios. As such, we have built InDelible to be scalable to many thousands of samples (Figure S2).

Other studies have previously noted that ∼10% of all *MECP2* variants in probands ascertained based on presentation of Rett-associated phenotypes were deletions^31^^,^^34^ and a large number of pathogenic or likely pathogenic variants in ClinVar fall within the same region of *MECP2* that we detail in this manuscript. These observations, combined with the diverse phenotypes that this study has identified (Figure 3B), further complicate the clinical interpretation of variants disrupting *MECP2*. In particular, the work of Guy et al.^29^ found that slight differences between the size and sequence context of deletions in the C-terminal domain of *MECP2* can have significant ramifications in RNA/protein expression. Additionally, Huppke et al.^35^ found that skewed X-inactivation could play a role in the severity of *MECP2* presentation. Further work is needed to understand how different classes of mutation lead to diverse phenotypes in individuals with *MECP2* loss-of-function variants. However, most importantly and exemplifying the additive power of InDelible, if not applied to the DDD study, 20.6% of DDD probands with clinically relevant *MECP2* variants would not have received a diagnosis for their disorder.

InDelible was designed to detect variant breakpoints missed by other approaches in ES data from individuals with DDs. This has three major ramifications for the design of InDelible and the variants discussed as part of this study. First, as the primary cause of DDs is highly penetrant dominant *de novo* variants,^7^ InDelible variant discovery was focused on identifying such variants from a defined list of genes known to be associated with DDs.^22^ As briefly demonstrated above for rare inherited variation, this does not preclude the use of InDelible to identify variants acting through other modes of inheritance; InDelible will identify variants across the entire allele frequency spectrum and outside of the provided gene list as part of the primary output.

**Figure 4:**
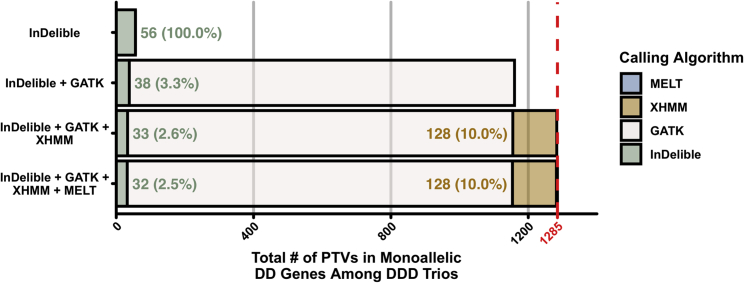
Added diagnostic PTV yield of InDelible Total number of *de novo* PTVs (y axis) ascertained in DD-associated genes when using InDelible alone, or in combination with a subset of three additional algorithms (GATK,^11^ XHMM,^9^ or MELT^3^^,^^36^). Percentages represent the proportion of all PTVs specific to InDelible (green text) or XHMM (orange text) for each bar. The red line and axis label indicates the maximum number of *de novo* PTVs identified in DD-associated genes among 9,848 DDD trio probands if combining data from all four algorithms (n = 1,285 variants).

Second, the DDD cohort has been previously investigated for a broader range of variant classes (using both different assays and algorithms) than most ES studies. For ES-based CNV discovery from read-depth, DDD applied four separate algorithms to build a joint call set (unpublished data). Thus, the added diagnostic value of running InDelible is probably under-estimated in the DDD study compared to other ES studies and/or common clinical sequencing practices which would be unlikely to utilize complex joint-calling approaches such as our own. To quantify the added diagnostic value of running InDelible across different settings by a user seeking to run a minimal number of algorithms, we estimated the proportion of unique PTVs InDelible would find if used alone or jointly with other algorithms targeting a breadth of variant types (SNVs, indels, large deletions, and MEIs; supplemental material and methods).^3^^,^^9^^,^^11^ Overall, and when using other approaches, InDelible-specific variants will likely represent between 2%–3% of all PTVs in a given cohort (Figure 4). This observation strongly implies that workflows that do not incorporate algorithms capable of detecting this class of cryptic variation are likely to achieve only 97%–98% sensitivity for pathogenic PTVs.

Finally, we note that InDelible is unlikely to be more effective than currently available tools when applied to genome-sequencing data. In ES, discordant read pairs are typically much less informative for detecting SVs than in genome sequencing due to the inherent properties of the data. In genome sequencing, data combining split and discordant read-pair information is a better means to identify most SV types.

InDelible provides a rapid framework for the assessment of ES data for intermediate-length pathogenic SVs of diverse mutational origins. Our results show that through a combination of enhanced algorithm design, variant annotation, and clinical interpretation, ongoing interrogation of well-studied datasets will continue to yield improved diagnoses.

## Data and code availability

Sequencing, phenotype data, and variant calls for all data in this paper are accessible via the European Genome-phenome Archive (EGA) under study EGAS00001000775. InDelible is available at the InDelible GitHub repository, https://github.com/HurlesGroupSanger/indelible. All code and data used to generate figures and results in this manuscript are located at the following GitHub repository: https://github.com/HurlesGroupSanger/indelible_paper. A Docker image of InDelible is available at the following GitHub repository: https://github.com/wtsi-hgi/indelible-docker/tree/master.

## Web resources

pysam: htslib interface for python, https://pysam.readthedocs.io/en/latest/

## Supplemental information

Document S1. Figures S1–S9, Table S1, and supplemental material and methods

Table S2. *De novo* variants or variants of unknown inheritance identified as part of this study

Table S3. Phenotypes of probands with *MECP2* structural variants

Table S4. Inherited variants identified by InDelible

Document S2. Article plus supplemental information

## References

[1] Collins R.L., Brand H., Karczewski K.J., Zhao X., Alföldi J., Francioli L.C., Khera A.V., Lowther C., Gauthier L.D., Wang H., Genome Aggregation Database Production Team, Genome Aggregation Database Consortium (2020). A structural variation reference for medical and population genetics. Nature.

[2] Zhao X., Collins R.L., Lee W.-P., Weber A.M., Jun Y., Zhu Q., Weisburd B., Huang Y., Audano P.A., Wang H., Human Genome Structural Variation Consortium (2021). Expectations and blind spots for structural variation detection from long-read assemblies and short-read genome sequencing technologies. Am. J. Hum. Genet..

[3] Gardner E.J., Prigmore E., Gallone G., Danecek P., Samocha K.E., Handsaker J., Gerety S.S., Ironfield H., Short P.J., Sifrim A. (2019). Contribution of retrotransposition to developmental disorders. Nat. Commun..

[4] Sanchis-Juan A., Stephens J., French C.E., Gleadall N., Mégy K., Penkett C., Shamardina O., Stirrups K., Delon I., Dewhurst E. (2018). Complex structural variants in Mendelian disorders: identification and breakpoint resolution using short- and long-read genome sequencing. Genome Med..

[5] Torene R.I., Galens K., Liu S., Arvai K., Borroto C., Scuffins J., Zhang Z., Friedman B., Sroka H., Heeley J. (2020). Mobile element insertion detection in 89,874 clinical exomes. Genet. Med..

[6] Kosugi S., Momozawa Y., Liu X., Terao C., Kubo M., Kamatani Y. (2019). Comprehensive evaluation of structural variation detection algorithms for whole genome sequencing. Genome Biol..

[7] Kaplanis J., Samocha K.E., Wiel L., Zhang Z., Arvai K.J., Eberhardt R.Y., Gallone G., Lelieveld S.H., Martin H.C., McRae J.F., Deciphering Developmental Disorders Study (2020). Evidence for 28 genetic disorders discovered by combining healthcare and research data. Nature.

[8] Schwarze K., Buchanan J., Taylor J.C., Wordsworth S. (2018). Are whole-exome and whole-genome sequencing approaches cost-effective? A systematic review of the literature. Genet. Med..

[9] Fromer M., Moran J.L., Chambert K., Banks E., Bergen S.E., Ruderfer D.M., Handsaker R.E., McCarroll S.A., O’Donovan M.C., Owen M.J. (2012). Discovery and statistical genotyping of copy-number variation from whole-exome sequencing depth. Am. J. Hum. Genet..

[10] Srivastava S., Love-Nichols J.A., Dies K.A., Ledbetter D.H., Martin C.L., Chung W.K., Firth H.V., Frazier T., Hansen R.L., Prock L., NDD Exome Scoping Review Work Group (2019). Meta-analysis and multidisciplinary consensus statement: exome sequencing is a first-tier clinical diagnostic test for individuals with neurodevelopmental disorders. Genet. Med..

[11] McKenna A., Hanna M., Banks E., Sivachenko A., Cibulskis K., Kernytsky A., Garimella K., Altshuler D., Gabriel S., Daly M., DePristo M.A. (2010). The Genome Analysis Toolkit: a MapReduce framework for analyzing next-generation DNA sequencing data. Genome Res..

[12] Short P.J., McRae J.F., Gallone G., Sifrim A., Won H., Geschwind D.H., Wright C.F., Firth H.V., FitzPatrick D.R., Barrett J.C., Hurles M.E. (2018). De novo mutations in regulatory elements in neurodevelopmental disorders. Nature.

[13] Lord J., Gallone G., Short P.J., McRae J.F., Ironfield H., Wynn E.H., Gerety S.S., He L., Kerr B., Johnson D.S., Deciphering Developmental Disorders study (2019). Pathogenicity and selective constraint on variation near splice sites. Genome Res..

[14] Kaplanis J., Akawi N., Gallone G., McRae J.F., Prigmore E., Wright C.F., Fitzpatrick D.R., Firth H.V., Barrett J.C., Hurles M.E., Deciphering Developmental Disorders study (2019). Exome-wide assessment of the functional impact and pathogenicity of multinucleotide mutations. Genome Res..

[15] Liaw A., Wiener M. (2002). Classification and Regression by randomForest. R News.

[16] Li H., Durbin R. (2010). Fast and accurate long-read alignment with Burrows-Wheeler transform. Bioinformatics.

[17] Camacho C., Coulouris G., Avagyan V., Ma N., Papadopoulos J., Bealer K., Madden T.L. (2009). BLAST+: architecture and applications. BMC Bioinformatics.

[18] Li H., Handsaker B., Wysoker A., Fennell T., Ruan J., Homer N., Marth G., Abecasis G., Durbin R., 1000 Genome Project Data Processing Subgroup (2009). The Sequence Alignment/Map format and SAMtools. Bioinformatics.

[19] Chen X., Schulz-Trieglaff O., Shaw R., Barnes B., Schlesinger F., Källberg M., Cox A.J., Kruglyak S., Saunders C.T. (2016). Manta: rapid detection of structural variants and indels for germline and cancer sequencing applications. Bioinformatics.

[20] Zook J.M., Catoe D., McDaniel J., Vang L., Spies N., Sidow A., Weng Z., Liu Y., Mason C.E., Alexander N. (2016). Extensive sequencing of seven human genomes to characterize benchmark reference materials. Sci. Data.

[21] Zook J.M., Hansen N.F., Olson N.D., Chapman L., Mullikin J.C., Xiao C., Sherry S., Koren S., Phillippy A.M., Boutros P.C. (2020). A robust benchmark for detection of germline large deletions and insertions. Nat. Biotechnol..

[22] Thormann A., Halachev M., McLaren W., Moore D.J., Svinti V., Campbell A., Kerr S.M., Tischkowitz M., Hunt S.E., Dunlop M.G. (2019). Flexible and scalable diagnostic filtering of genomic variants using G2P with Ensembl VEP. Nat. Commun..

[23] Thorvaldsdóttir H., Robinson J.T., Mesirov J.P. (2013). Integrative Genomics Viewer (IGV): high-performance genomics data visualization and exploration. Brief. Bioinform..

[24] Karczewski K.J., Francioli L.C., Tiao G., Cummings B.B., Alföldi J., Wang Q., Collins R.L., Laricchia K.M., Ganna A., Birnbaum D.P., Genome Aggregation Database Consortium (2020). The mutational constraint spectrum quantified from variation in 141,456 humans. Nature.

[25] Wright C.F., McRae J.F., Clayton S., Gallone G., Aitken S., FitzGerald T.W., Jones P., Prigmore E., Rajan D., Lord J., DDD Study (2018). Making new genetic diagnoses with old data: iterative reanalysis and reporting from genome-wide data in 1,133 families with developmental disorders. Genet. Med..

[26] Amir R.E., Van den Veyver I.B., Wan M., Tran C.Q., Francke U., Zoghbi H.Y. (1999). Rett syndrome is caused by mutations in X-linked MECP2, encoding methyl-CpG-binding protein 2. Nat. Genet..

[27] Lek M., Karczewski K.J., Minikel E.V., Samocha K.E., Banks E., Fennell T., O’Donnell-Luria A.H., Ware J.S., Hill A.J., Cummings B.B., Exome Aggregation Consortium (2016). Analysis of protein-coding genetic variation in 60,706 humans. Nature.

[28] Neul J.L., Kaufmann W.E., Glaze D.G., Christodoulou J., Clarke A.J., Bahi-Buisson N., Leonard H., Bailey M.E.S., Schanen N.C., Zappella M., RettSearch Consortium (2010). Rett syndrome: revised diagnostic criteria and nomenclature. Ann. Neurol..

[29] Guy J., Alexander-Howden B., FitzPatrick L., DeSousa D., Koerner M.V., Selfridge J., Bird A. (2018). A mutation-led search for novel functional domains in MeCP2. Hum. Mol. Genet..

[30] Watson P., Black G., Ramsden S., Barrow M., Super M., Kerr B., Clayton-Smith J. (2001). Angelman syndrome phenotype associated with mutations in MECP2, a gene encoding a methyl CpG binding protein. J. Med. Genet..

[31] Bebbington A., Percy A., Christodoulou J., Ravine D., Ho G., Jacoby P., Anderson A., Pineda M., Ben Zeev B., Bahi-Buisson N. (2010). Updating the profile of C-terminal MECP2 deletions in Rett syndrome. J. Med. Genet..

[32] Wright C.F., Fitzgerald T.W., Jones W.D., Clayton S., McRae J.F., van Kogelenberg M., King D.A., Ambridge K., Barrett D.M., Bayzetinova T., DDD study (2015). Genetic diagnosis of developmental disorders in the DDD study: a scalable analysis of genome-wide research data. Lancet.

[33] Ye K., Schulz M.H., Long Q., Apweiler R., Ning Z. (2009). Pindel: a pattern growth approach to detect break points of large deletions and medium sized insertions from paired-end short reads. Bioinformatics.

[34] Krishnaraj R., Ho G., Christodoulou J. (2017). RettBASE: Rett syndrome database update. Hum. Mutat..

[35] Huppke P., Maier E.M., Warnke A., Brendel C., Laccone F., Gärtner J. (2006). Very mild cases of Rett syndrome with skewed X inactivation. J. Med. Genet..

[36] Gardner E.J., Lam V.K., Harris D.N., Chuang N.T., Scott E.C., Pittard W.S., Mills R.E., Devine S.E., 1000 Genomes Project Consortium (2017). The Mobile Element Locator Tool (MELT): population-scale mobile element discovery and biology. Genome Res..

