# Supplementary material for: Detecting cryptic clinically relevant structural variation in exome-sequencing data increases diagnostic yield for developmental disorders
Source: Am J Hum Genet. 2021 Oct 8;108(11):2186–94. doi: 10.1016/j.ajhg.2021.09.010 (PMC8595893; doi:10.1016/j.ajhg.2021.09.010)
Supplement: Document S2. Article plus supplemental information [file mmc5.pdf]

REPORT

Detecting cryptic clinically relevant structural variation in exome-sequencing data increases diagnostic yield for developmental disorders

Eugene J. Gardner,[1,6] Alejandro Sifrim,[2] Sarah J. Lindsay,[1] Elena Prigmore,[1] Diana Rajan,[1] Petr Danecek,[1] Giuseppe Gallone,[1] Ruth Y. Eberhardt,[1] Hilary C. Martin,[1] Caroline F. Wright,[3] David R. FitzPatrick,[4] Helen V. Firth,[1,5] and Matthew E. Hurles[1,*]

Summary

Structural variation (SV) describes a broad class of genetic variation greater than 50 bp in size. SVs can cause a wide range of genetic diseases and are prevalent in rare developmental disorders (DDs). Individuals presenting with DDs are often referred for diagnostic testing with chromosomal microarrays (CMAs) to identify large copy-number variants (CNVs) and/or with single-gene, gene-panel, or exome sequencing (ES) to identify single-nucleotide variants, small insertions/deletions, and CNVs. However, individuals with pathogenic SVs undetectable by conventional analysis often remain undiagnosed. Consequently, we have developed the tool InDelible, which interrogates short-read sequencing data for split-read clusters characteristic of SV breakpoints. We applied InDelible to 13,438 probands with severe DDs recruited as part of the Deciphering Developmental Disorders (DDD) study and discovered 63 rare, damaging variants in genes previously associated with DDs missed by standard SNV, indel, or CNV discovery approaches. Clinical review of these 63 variants determined that about half (30/63) were plausibly pathogenic. InDelible was particularly effective at ascertaining variants between 21 and 500 bp in size and increased the total number of potentially pathogenic variants identified by DDD in this size range by 42.9%. Of particular interest were seven confirmed *de novo* variants in *MECP2*, which represent 35.0% of all *de novo* protein-truncating variants in *MECP2* among DDD study participants. InDelible provides a framework for the discovery of pathogenic SVs that are most likely missed by standard analytical workflows and has the potential to improve the diagnostic yield of ES across a broad range of genetic diseases.

Structural variation (SV) includes a diverse collection of genomic rearrangements such as copy number variation (CNV), mobile element insertions (MEIs), inversions, translocations, and others.[1] Depending on population ancestry and technology used, the typical human genome harbors between 7,000 and 25,000 polymorphic SVs, with the majority constituting bi-allelic CNVs and MEIs.[2] While most SVs have minimal, if any, functional impact, SVs have been recognized as causative variants in congenital disorders.[3–5]

In diagnostic testing of suspected genetic disorders, SVs are often identified using chromosomal microarrays (CMAs) which offer a low-cost albeit low-resolution method for the identification of large CNVs (typically >20 kbp in length for genic regions). CMAs are still widely used by diagnostic laboratories despite the increasing maturity of genome sequencing-based tools for SV discovery[6] and the wealth of clinically ascertained exome-sequencing (ES) data already generated for the ascertainment of single-nucleotide variants (SNVs) and small insertions/deletions (indels).[7] There are several reasons for this. First, the cost, computational power, and informatics complexity necessary for genome sequencing-based diagnostics is still a barrier to many public and private healthcare providers.[8] Second, current ES-based SV-discovery approaches focus on methods that interrogate sequencing coverage to identify regions of copy number variation within one genome compared to others.[9] As such, ascertainment is typically limited to CNVs of size >10 kbp, with resolution largely a factor of the sequencing depth and the density and number of baits in the ES assay, analogous to probes in CMAs. Thus, despite potentially offering improvements in CNV ascertainment over CMAs, ES as a tool for the assessment of diagnostic SVs has been slow to enter the clinic.[10]

Consequently, individuals with genetic abnormalities smaller than the discovery resolution of CMA or standard SV-ES approaches (>10 kbp) but larger than variants able to be accurately called using typical SNV/indel-based approaches (<50 bp)[11] often remain undetected, here termed "cryptic." To address this unmet need, we have developed the tool InDelible, which examines ES data for split read pairs indicative of SV breakpoints. We decided to focus on split reads because the formation of unique junction sequences is a shared characteristic of a broad range of different classes of SVs. We applied InDelible to ES data

[1]Wellcome Sanger Institute, Wellcome Genome Campus, Cambridge, Hinxton CB10 1SA, UK; [2]Department of Human Genetics, KU Leuven, Herestraat 49, Box 602, Leuven 3000, Belgium; [3]University of Exeter Medical School, Institute of Biomedical and Clinical Science, Royal Devon and Exeter Hospital, Exeter EX2 5DW, UK; [4]MRC Human Genetics Unit, Institute of Genetics and Cancer, University of Edinburgh, WGH, Edinburgh EH4 2SP, UK; [5]East Anglian Medical Genetics Service, Box 134, Cambridge University Hospitals NHS Foundation Trust, Cambridge Biomedical Campus, Cambridge CB2 0QQ, UK
[6]Present address: Medical Research Council (MRC) Epidemiology Unit, University of Cambridge School of Clinical Medicine, Institute of Metabolic Science, Cambridge Biomedical Campus, Cambridge CB2 0QQ, UK
*Correspondence: meh@sanger.ac.uk
https://doi.org/10.1016/j.ajhg.2021.09.010.
© 2021 The Authors. This is an open access article under the CC BY license (http://creativecommons.org/licenses/by/4.0/).

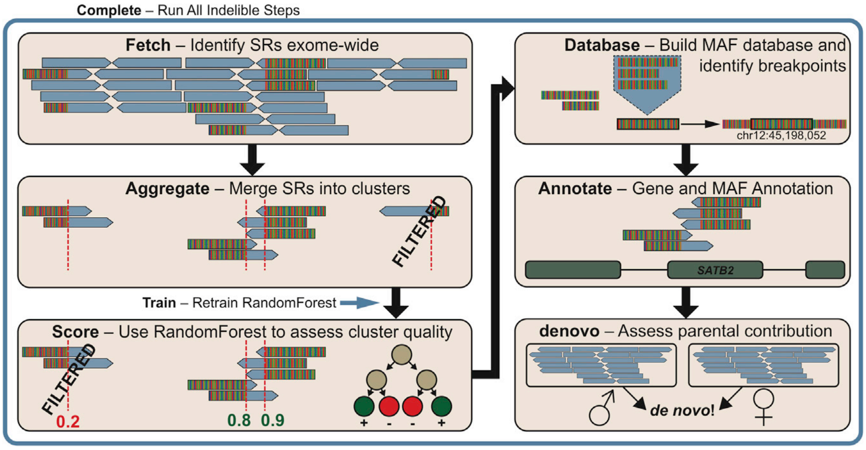

Figure 1. InDelible SV discovery in ES data
InDelible processes one ES sample provided in BAM or CRAM format via six primary steps (tan boxes). First, alignment files are queried for all reads where part of the aligned sequence matches the reference genome and the other does not (i.e., split reads; Fetch). Next, reads are clustered (Aggregate) and scored using a random forest model[15] trained using a variant truth set (Score; see Figure S1 and supplemental material and methods for more detail). Split reads are then merged within clusters across individuals to determine the longest quality junction sequence and mapped back to the genome with bwa mem[16] and to a set of curated repeats with blastn.[17] These alignments are then used to determine breakpoint frequency, likely breakpoints, length, and structural variant class (i.e., deletion, duplication, insertion, etc.; Database). Split read clusters are subsequently annotated with population frequency and intersection with genomic functional annotations, such as protein-coding genes (Annotate). Finally, clusters are assessed for presence or absence in parental samples, where available, to determine inheritance status and identify likely *de novo* variants (denovo). All of these commands can be run on one sample via the "Complete" command (blue box). InDelible also includes the "Train" command to train a new random forest model from user-provided labeled training data.

generated from 13,438 probands with severe developmental disorders (DDs) recruited as part of the Deciphering Developmental Disorders (DDD) study. Approximately 29% of DDD probands harbor a pathogenic *de novo* mutation in a gene known to be associated with DD[7] and have been previously assessed for a wide range of variant classes such as coding,[7] noncoding,[12] and splice site[13] SNVs and indels, multinucleotide variants,[14] mobile element insertions,[3] and copy number variants (unpublished data). As such, the DDD study represents an ideal opportunity to demonstrate the additive diagnostic potential of identification of SVs at scale using split-read information.

InDelible variant discovery and analysis proceeds in several steps (Figure 1; detailed description in supplemental material and methods). In summary, InDelible identifies split reads, aggregates them into clusters at the same genomic location, filters these clusters to remove technical artifacts and retain likely genetic variants, and then combines unaligned portions of split reads and maps them to the genome to characterize the nature of the variant. InDelible also calculates the frequency of each split-read cluster across a population of individuals to facilitate the filtering of variants on the basis of minor allele frequency.

InDelible is coded in Python, uses the pysam (see web resources) library for sequence alignment file manipulation (Table S1), and works on bwa-aligned BAM or CRAM format files.[18] We have designed InDelible to be scalable for datasets comprising individual probands to multi-thousand sample cohorts and our estimates suggest that, to analyze a dataset of 1,000 trios, InDelible would require approximately 1556 CPU h, or 15.6 h of real time on a 100-core compute cluster (Figure S2). Additionally, for easy implementation on cloud compute platforms, we have made InDelible available as a Docker image (see supplemental material and methods and data and code availability).

We benchmarked InDelible against GATK[11] and Manta,[19] another SV detector which utilizes split reads, for variants across a range of allele frequencies and sizes. First, we ran these callers on ES data generated for a control individual by the Genome in a Bottle Consortium.[20,21] We then used the gold-standard variant dataset provided by the Genome in a Bottle Consortium for the same individual, which amalgamates variant-call data across several data types including whole genome short-, linked-, and long-read sequencing, to assess recall and specificity of the resulting ES calls for each algorithm (Figure S3; supplemental material and methods). When using ES data, InDelible equals or exceeds the recall of both GATK and Manta for variants between 21 and 10 kbp in length, the variant space InDelible was targeted to identify. Relative to InDelible, GATK and Manta had 81.7% and 15.0% recall for deletions >20 bp in length, respectively, and 86.9% and 8.2% recall for insertions >20 bp in length, respectively. In this same experiment InDelible has moderately increased false discovery rates compared to GATK (Figure S3). These issues can likely be attributed to InDelible being designed for maximum sensitivity in clinical sequencing data and can likely be abrogated via the design of better hard filters when analyzing population-level variants and/or retraining the random forest using training data from population-level datasets.

A key objective for the design of InDelible was to identify *de novo* variants potentially causative of a proband's disorder. As such, variants are primarily filtered on: (1) the population frequency of the split read cluster to remove variants too common to be plausibly causative of a rare disorder, (2) absence in unaffected parents (when available), and (3) intersection of variant breakpoints with the coding

sequences of known-disease-associated genes. Defining the precise molecular structure of SVs from short read sequencing data can be challenging, and even minor errors in breakpoint precision can have large consequences on interpretation (e.g., in- versus out-of-frame indels). Hence, we opted to identify all variants which intersect relevant DD-associated genes for further manual curation rather than relying on generic variant interpretation tools.

To evaluate the utility of InDelible for diagnostic analyses, we applied InDelible to identify putatively diagnostic variants in 13,438 probands recruited to the DDD study. Probands were exome sequenced either with both parents (trios, n = 9,848) or with one or both parents absent (non-trios, n = 3,590). We first identified split reads and split read clusters (Figure 1) to ascertain 353,313,108 redundant split read clusters across all probands. Random forest filtering resulted in retention of 30,667,420 high-quality, redundant split read clusters across all probands, or 8.7% of originally ascertained loci (supplemental material and methods, Figure S4). After cluster filtering, we merged all retained clusters into a set of 1,954,642 non-redundant split read clusters across all 13,438 probands, with 1,342,050 (68.7%) clusters found only in one proband (Figure S5). Clusters were evenly distributed across all chromosomes as a function of chromosome length ($r^2 = 0.739$; Figure S6). Retained clusters were then annotated with putative breakpoints, intersecting gene(s), and population frequency. InDelible was also able to determine the missing 5′ or 3′ breakpoint, variant length, and variant type (i.e., deletion, duplication, MEI, etc.) of 199,932 (10.2%) clusters (supplemental material and methods). Of the clusters which InDelible was able to resolve to a specific variant type, 65.7% were simple deletions/duplications, with the remainder comprising complex events, MEIs, translocations/segmental duplications, and non-templated insertions (Figure S7). Ascertainment of variant type and length are dependent on sequencing depth and population frequency (Figure S5) but are optimized for the length of variants InDelible is best suited to identify (~20–500 bp; Figure S8). This specificity is best demonstrated when restricting to clusters that are plausibly associated with DDD study participant phenotype (see below); InDelible accurately resolves both breakpoints, length, and variant type for 86.3% (126/146) of such clusters (Table S2).

We next restricted our variant set to rare (call frequency < 0.04%) clusters found only in or near (here defined as within ±10 bp of any exon) the coding sequence of 399 dominant or X-linked DD-associated genes from the Developmental Disorders Genotype-to-Phenotype database (DDG2P).[22] Variants identified within individuals sequenced as a parent-offspring trio were then also assessed for *de novo* status. Filtering on allele frequency, inheritance, and gene intersection resulted in a preliminary set of 260 candidate indels and SVs across all 13,438 probands (Figure 2A; Table S2; supplemental material and methods). Based on manual variant inspection,[23] we determined that 2/260 (0.8%) were erroneously annotated to have intersected a mono-allelic DD gene, 17/260 (6.5%) candidate *de novo* events were likely to be present in a parent (i.e., parental false negatives), and 23/260 (8.8%) were unlikely to be real variants (i.e., offspring false positives). Four probands contributed 52.2% of false positive variants, indicating that sample selection and/or additional sample-level QC could further lower the false positive rate of InDelible (Figure 2A; Table S2).

Following variant quality control, we further curated variants for those likely to be associated with a proband phenotype (Figure 2A). We considered variants with a non-Finnish European minor allele frequency of $\geq 1 \times 10^{-4}$ (19/260; 7.3%) in the Genome Aggregation Database (gnomAD)[1,24] or presence in other unrelated individuals within DDD (20/260; 7.7%) as unlikely to be the cause of the child's disorder. Additionally, variants confined to introns or 5′/3′ UTRs were also defined as variants of uncertain significance and were not considered further (33/260; 12.7%). This final round of filtering left 146 SVs and large indels which could plausibly explain a proband phenotype (56 from probands sequenced as trios, 90 from non-trio probands).

We next sought to determine the sensitivity of InDelible to clinically relevant variants ascertained using alternative methods. DDD has already identified (across both trio and non-trio probands) 1,853 rare, plausibly pathogenic variants with a net size difference ≥1 bp (i.e., non-SNVs) in the same DDG2P gene set defined above[7]—variants potentially detectable with a split read-based method such as that employed by InDelible. The majority of these variants are private or low-allele frequency small indels between 1 and 10 bp in size (1,218/1,853; 65.7%) or large CMA or ES-ascertained CNVs ≥10 kbp in length (410/1,853; 22.1%; Figure 2B). As anticipated due to the low number of split reads at variant breakpoints as variant size decreases, InDelible performed poorly in identification of very short variants ≤10 bp with an overall sensitivity of 1.4% (Figure 2C). Sensitivity improved as a function of variant size, peaking at 48.3% sensitivity for variants between 21 and 50 bp, but dropped again for variants ≥100 bp. To better understand why InDelible missed such variants, we manually curated the 34 potentially pathogenic variants between 21 and 500 bp not identified by InDelible. We found that InDelible missed variants for three primary reasons. First, these potentially pathogenic variants include some higher-frequency variants that are too common to be plausibly pathogenic whose true allele frequency was underestimated previously, but have now been more accurately determined by InDelible and thus subsequently filtered out (n = 12/34; 35.3%). Second, several variants have low split read support (i.e., <5 reads) despite being located in high-coverage regions and were thus excluded by our stringent filtering approach (n = 11/34; 32.4%). Third, as variant size increases, it becomes more likely that the breakpoints of SVs which impact coding sequence lie outside of ES target regions (i.e., within intronic and intergenic sequences).

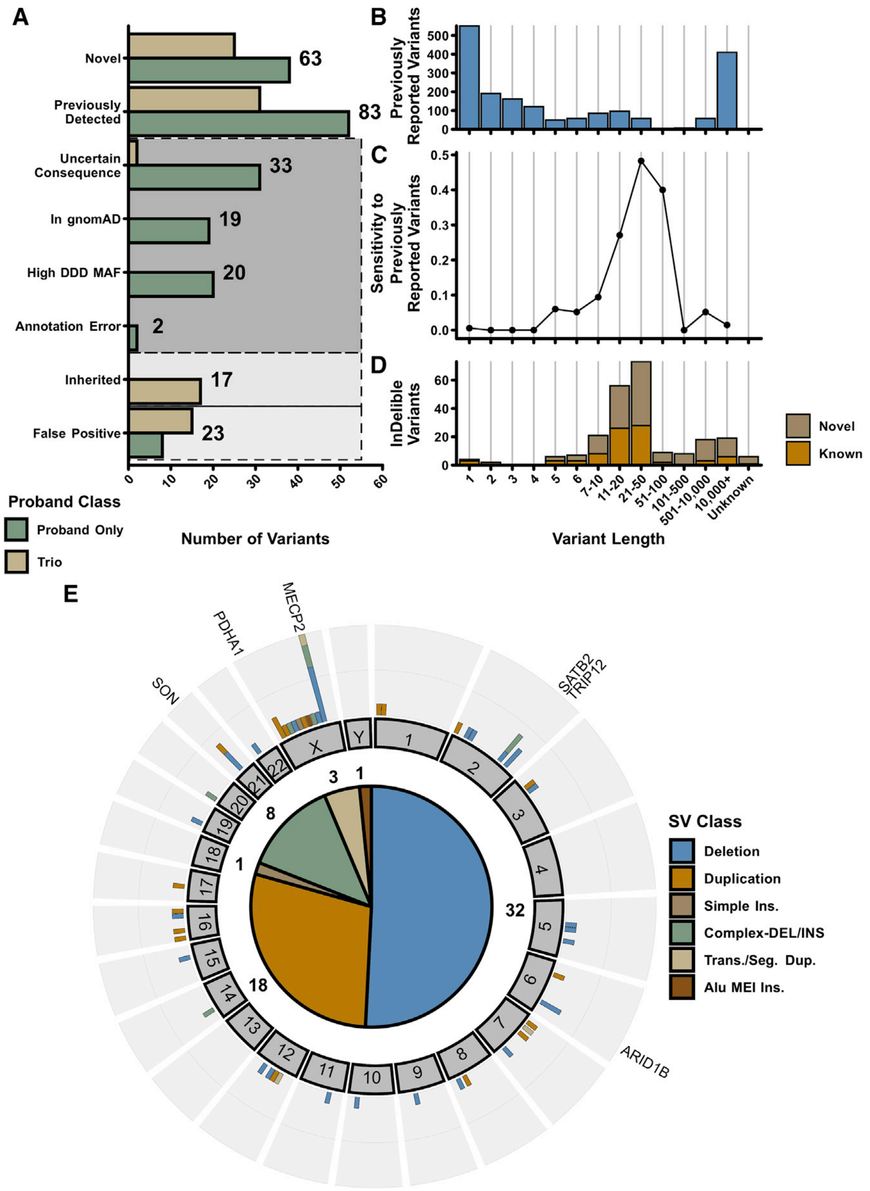

Figure 2. SV ascertainment in the DDD study with InDelible

(A) Breakdown of putative variant consequences for all 260 variants identified in this study delineated by whether or not the proband was sequenced with both parents (trio, tan) or not (proband only, dark green). Light gray and dark gray boxes represent erroneous variants and variants unlikely to be associated with a proband phenotype, respectively.

(B) Total number of DDD variants reported to referring clinicians via the DECIPHER platform among DDD probands with a net size change ≥ 1 bp.

(C) Sensitivity of InDelible to DDD variants reported to referring clinicians via the DECIPHER platform among various variant size bins.

(D) Categorization of InDelible-ascertained variants into previously known (orange) versus those novel (brown) to this study based on size.

(E) Distribution of variants unique to InDelible throughout the genome. Shown in the outer plot are the total number of InDelible variants per gene, with genes that have multiple previously undetected variants labeled. Displayed in the inner plot are the total number of variants for each SV type identified.

Ergo, such variants are refractory to identification with split reads and likely to be missed by any split-read caller (n = 6/34; 17.6%). Combined, these three explanations account for 85.3% of variants between 21 and 500 bp missed by InDelible. While variants with breakpoints outside of sequencing baits are invisible to InDelible, additional fine-tuning of InDelible's filtering parameters could, in theory, output variants with lower split read support or variants with higher allele frequencies.

These 63 previously undetected variants (four of which were ascertained by an earlier version of InDelible and included as part of a previous DDD publication[25]) that impact known DD-associated genes (Table S2) are composed primarily of deletions and duplications (50/63; 79.4%) but also includes variants with diverse mutational mechanisms such as MEIs, complex rearrangements, and dispersed duplications/translocations (Figure 2E). 25 of these variants were observed in trio probands, with parental data supporting a *de novo* origin for all of these variants. InDelible was particularly effective at identifying variants between 21 and 500 bp in size (Figure 2D); 30 previously undetected variants (47.6% of InDelible-specific variants) lie within this size range and represent a 42.9% increase in putatively pathogenic variants 21–500 bp in length among DDD probands (Figure 2D). We also identified six genes with multiple previously undetected SVs among unrelated individuals, of which the most recurrently affected was *MECP2,* the causal gene of Rett syndrome (Figure 2E).[26]

From an initial round of clinical review, based on intersecting gene(s) and associated phenotypes, we concluded that nine (14.3%) of these 63 previously undetected variants were unlikely to explain the referred proband's phenotype, and were thus excluded from future analysis (Table S2). We next attempted PCR validation of the 54 putatively pathogenic variants (supplemental material and methods). Of the variants for which conclusive validation results could be obtained, 23/23 (100%) were confirmed as true positives, either by the obvious presence of a mutant band of expected size with gel electrophoresis or by follow-up capillary sequencing where the gel result was uncertain (Table S2). For variants for which PCR was possible, we also confirmed that 10/10 (100%) putative *de novo*

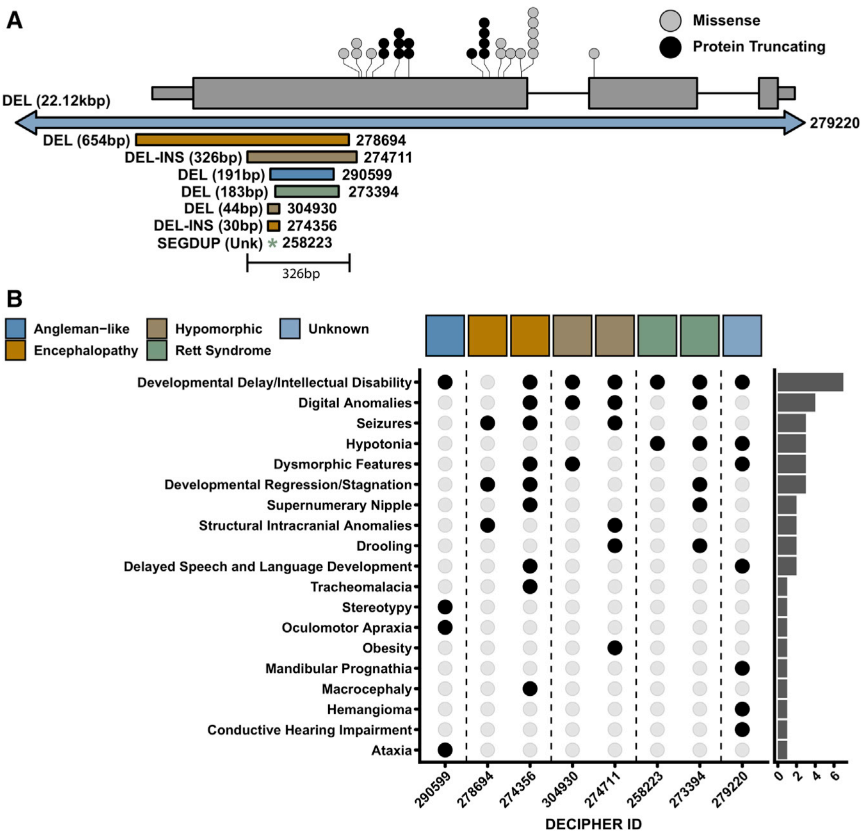

Figure 3. Clustered SVs in MECP2 cause diverse phenotypes

(A) Shown is a cartoon representation of the gene *MECP2*, with stop-gained (black circles) and missense (gray circles) *de novo* SNVs identified in DDD trios. Each circle represents one proband, with recurrent variants represented by stacks of circles. Below the *MECP2* gene model, we have shown the seven variants identified by InDelible as well as the single whole gene deletion previously identified via CMA (proband 279220; arrows indicate this variant extends beyond the scale shown in the diagram). Sizes adjacent to variants represent the difference in number of reference and alternate bases in the indicated DDD study participant genome. We have indicated that the variant in DDD study participant 258223 only incorporates non-references bases (i.e., an insertion) with an asterisk. Variants are colored by their classification in (B). All InDelible-ascertained variants overlap the same 326 bp region in the last exon of *MECP2*.

(B) Diverse proband phenotypes among *MECP2* SV carriers. Each proband carrying a *MECP2* SV from (A) is shown on the x axis, with phenotypes annotated by the referring clinician shown on the y axis. Filled black circles represent when a corresponding proband displays the corresponding phenotype. Colored boxes on the top of the plot represent the diverse phenotypes we identified following clinical review. The y axis marginal histogram represents the number of times the corresponding phenotype was observed among our SV probands.

variants identified in trio probands were indeed absent from both parents.

All 54 plausible pathogenic variants were reported to referring clinicians and clinically interpreted by two senior clinical geneticists; 30/54 (55.6%) were classified as pathogenic or likely pathogenic by both clinical geneticists (Table S2). Of these variants, those identified in non-trio probands ($n = 31/54$ plausibly pathogenic variants) for which inheritance status is unavailable, were less likely to be interpreted as being pathogenic (Fisher's $p = 0.006$). This finding is corroborated by the difference in the proportion of in-frame versus out-of-frame deletions and duplications ≤ 50 bp between trio and non-trio probands; 80.0% of deletions and duplications are in-frame for non-trios versus 19.0% for trios (Fisher's $p = 1.5 \times 10^{-6}$; Figure S9). This is consistent with population-level observations: out-of-frame deletions and duplications are typically under stronger negative selection than in-frame variants[27] and an increased proportion of in-frame variants in non-trio probands is suggestive of a greater proportion being benign. The difference is likely attributable to the absence of parental data leading to the inclusion of rare benign inherited variants that are unlikely to be filtered out using population variation data (e.g., gnomAD[1,24]). Overall, *de novo* variants identified by InDelible represent 0.7% (18/2592) of all confirmed diagnoses among trio probands in the DDD study.

InDelible identified a total of seven confirmed *de novo* variants ≥ 20 bp in length affecting *MECP2* (Figures 2E and 3A), all predicted to be protein truncating. As expected and in accordance with known sex bias among individuals with Rett syndrome,[28] all variants were ascertained from female probands. Out of these seven probands, two have phenotypes that could be described as consistent with typical Rett syndrome presentation.[28] Through in-depth clinical curation of HPO terms (see supplemental material and methods), we grouped probands with putative loss-of-function mutations caused by SVs in *MECP2* into four categories (Figure 3B). Cases identified by InDelible thus represent the wide variety of diverse clinical presentations that can result from disruption of the C terminus of *MECP2*[29] and include previously observed *MECP2*-associated phenotypes such as early-onset seizures and Angelman-like symptoms (Table S3; Figure 3B).[30]

Interestingly, all five of our *MECP2* variants in probands without typical Rett syndrome presentation overlapped the same 326 bp region located within the final coding exon and, aside from a previously ascertained whole

gene deletion (proband 279220), do not overlap with putatively pathogenic SNVs identified within the DDD study (Figure 3A). The SV-specific region corresponds to an area of low sequence complexity and has been previously ascertained as hyper-mutable by several studies.[29,31] The molecular function of this region of *MECP2* is poorly understood and it is uncertain as to the consequences that our described variants may have on protein structure beyond decreasing transcript abundance and/or overall protein stability.[29]

The seven *de novo MECP2* variants constitute 28.0% (7/25) of all novel *de novo* variants identified by InDelible and 35.0% (7/20) of all confirmed *de novo* protein-truncating or gene-deleting variants of *MECP2* in the DDD study[7] (Figure 3A).

As several publications have shown that rare, inherited variants are also important in the genetic architecture of developmental disorders,[32] we next sought to examine whether InDelible could be used to identify such variants. We repeated our filtering as described above but limited to variants found in only a single proband with split read support from either parent (supplemental material and methods). This approach identified a total of 145 variants within the coding sequence of mono-allelic DD genes. As expected based on our analysis of variants in probands sequenced without their parents (Figure S9), a large proportion of inherited variants we identified were balanced/in-frame deletions or duplications with uncertain effect on the target protein (50; 34.5%). Others either primarily overlapped noncoding sequence, were found in an individual with a more likely diagnostic variant, were large duplications which only partially overlapped the gene of interest, were already identified based on an alternate breakpoint as part of our *de novo* analysis, or were also identified in control individuals at high enough allele frequencies to be considered unlikely to be associated with an individual's phenotype.[1,24] Initial filtering based on these criteria left a remainder of 17 variants for clinical interpretation.

Of the remaining inherited variants, seven were already identified via other approaches and reported to referring clinicians with six considered as likely benign and one as likely pathogenic. The remaining ten variants were referred to the same two senior clinician geneticists as for our *de novo* analysis detailed above (Table S4). Of these ten variants, all but one were unlikely to be involved in individual phenotype. The sole remaining inherited variant, an out-of-frame deletion in *KAT6B*, was identified in a proband-mother pair and was deemed a variant of uncertain consequence upon initial clinical review. Follow-up with the referring clinician regarding the mother's phenotype determined that the mother did not exhibit any features of the proband's disorder. As such, this variant was deemed to be likely benign. Combined, these data show that InDelible is effective at identifying rare, inherited variants but that the overall diagnostic yield may be low.

Here we present the development and application of InDelible, a tool designed for the rapid assessment of ES data for breakpoints of rare, pathogenic cryptic SVs involved in single-gene disorders (Figure 1). We applied InDelible to 13,438 proband genomes sequenced as part of the DDD study and identified a total of 146 candidate pathogenic variants impacting genes associated with dominant or X-linked DD (Figure 2A, Table S2). Of these 146 variants, 63 were not previously identified in DDD probands, despite the wide range of SV and InDel detection algorithms that have previously been deployed on this cohort.[7,25] Notably, we increased the number of putatively diagnostic variants among DDD probands 21–500 bp in length by 42.9% (Figure 2D). Through conservative clinical assessment of these 63 variants, we determined that 30 (47.6%) of our previously undetected variants were considered likely causative of proband phenotype—of particular interest was the large number of protein-truncating SVs we identified in *MECP2* (Figure 3).

The variant size range which InDelible interrogates is complementary to other approaches commonly used for variant discovery from ES data.[9,11] While other previously described algorithms have also attempted to mine split read information for structural variant detection,[11,19,33] they have different properties that preclude meaningful comparison with InDelible.[11] Some have been trained primarily on genome sequencing data rather than ES data,[19,33] others do not explicitly assess *de novo* status, and many are not readily scalable to a dataset of ~10,000 trios. As such, we have built InDelible to be scalable to many thousands of samples (Figure S2).

Other studies have previously noted that ~10% of all *MECP2* variants in probands ascertained based on presentation of Rett-associated phenotypes were deletions[31,34] and a large number of pathogenic or likely pathogenic variants in ClinVar fall within the same region of *MECP2* that we detail in this manuscript. These observations, combined with the diverse phenotypes that this study has identified (Figure 3B), further complicate the clinical interpretation of variants disrupting *MECP2*. In particular, the work of Guy et al.[29] found that slight differences between the size and sequence context of deletions in the C-terminal domain of *MECP2* can have significant ramifications in RNA/protein expression. Additionally, Huppke et al.[35] found that skewed X-inactivation could play a role in the severity of *MECP2* presentation. Further work is needed to understand how different classes of mutation lead to diverse phenotypes in individuals with *MECP2* loss-of-function variants. However, most importantly and exemplifying the additive power of InDelible, if not applied to the DDD study, 20.6% of DDD probands with clinically relevant *MECP2* variants would not have received a diagnosis for their disorder.

InDelible was designed to detect variant breakpoints missed by other approaches in ES data from individuals with DDs. This has three major ramifications for the design of InDelible and the variants discussed as part of this study. First, as the primary cause of DDs is highly penetrant dominant *de novo* variants,[7] InDelible variant discovery was

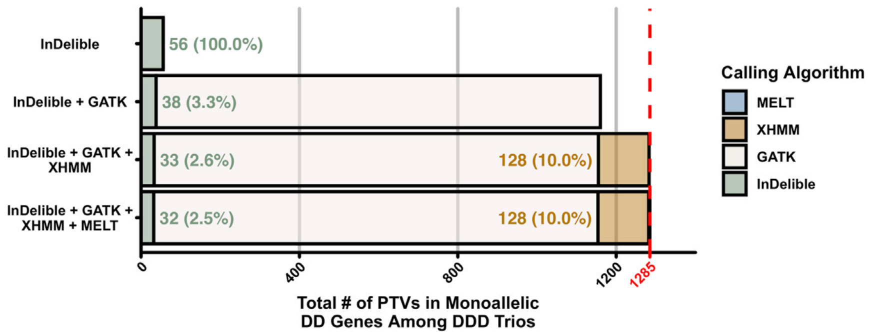

Figure 4. Added diagnostic PTV yield of InDelible
Total number of *de novo* PTVs (y axis) ascertained in DD-associated genes when using InDelible alone, or in combination with a subset of three additional algorithms (GATK,[11] XHMM,[9] or MELT[3,36]). Percentages represent the proportion of all PTVs specific to InDelible (green text) or XHMM (orange text) for each bar. The red line and axis label indicates the maximum number of *de novo* PTVs identified in DD-associated genes among 9,848 DDD trio probands if combining data from all four algorithms (n = 1,285 variants).

focused on identifying such variants from a defined list of genes known to be associated with DDs.[22] As briefly demonstrated above for rare inherited variation, this does not preclude the use of InDelible to identify variants acting through other modes of inheritance; InDelible will identify variants across the entire allele frequency spectrum and outside of the provided gene list as part of the primary output.

Second, the DDD cohort has been previously investigated for a broader range of variant classes (using both different assays and algorithms) than most ES studies. For ES-based CNV discovery from read-depth, DDD applied four separate algorithms to build a joint call set (unpublished data). Thus, the added diagnostic value of running InDelible is probably under-estimated in the DDD study compared to other ES studies and/or common clinical sequencing practices which would be unlikely to utilize complex joint-calling approaches such as our own. To quantify the added diagnostic value of running InDelible across different settings by a user seeking to run a minimal number of algorithms, we estimated the proportion of unique PTVs InDelible would find if used alone or jointly with other algorithms targeting a breadth of variant types (SNVs, indels, large deletions, and MEIs; supplemental material and methods).[3,9,11] Overall, and when using other approaches, InDelible-specific variants will likely represent between 2%–3% of all PTVs in a given cohort (Figure 4). This observation strongly implies that workflows that do not incorporate algorithms capable of detecting this class of cryptic variation are likely to achieve only 97%–98% sensitivity for pathogenic PTVs.

Finally, we note that InDelible is unlikely to be more effective than currently available tools when applied to genome-sequencing data. In ES, discordant read pairs are typically much less informative for detecting SVs than in genome sequencing due to the inherent properties of the data. In genome sequencing, data combining split and discordant read-pair information is a better means to identify most SV types.

InDelible provides a rapid framework for the assessment of ES data for intermediate-length pathogenic SVs of diverse mutational origins. Our results show that through a combination of enhanced algorithm design, variant annotation, and clinical interpretation, ongoing interrogation of well-studied datasets will continue to yield improved diagnoses.

Data and code availability

Sequencing, phenotype data, and variant calls for all data in this paper are accessible via the European Genome-phenome Archive (EGA) under study EGAS00001000775. InDelible is available at the InDelible GitHub repository, https://github.com/HurlesGroupSanger/indelible. All code and data used to generate figures and results in this manuscript are located at the following GitHub repository: https://github.com/HurlesGroupSanger/indelible_paper. A Docker image of InDelible is available at the following GitHub repository: https://github.com/wtsi-hgi/indelible-docker/tree/master.

Supplemental information

Supplemental information can be found online at https://doi.org/10.1016/j.ajhg.2021.09.010.

Acknowledgments

We would like to thank the DDD participants and their families—without their trust and confidence this work would not be possible. We also wish to acknowledge Jeffrey Barrett for his leadership role in the DDD study. The research team acknowledges the support of the National Institute for Health Research, through the Comprehensive Clinical Research Network. Informed and written consent was obtained for all families and the study was approved by the UK Research Ethics Committee (10/H0305/83, granted by the Cambridge South REC, and GEN/284/12 granted by the Republic of Ireland REC). The DDD study presents independent research commissioned by the Health Innovation Challenge Fund (grant number HICF-1009-003), a parallel funding partnership between Wellcome and the Department of Health, and the Wellcome Sanger Institute (grant number WT098051). The views expressed in this publication are those of the author(s) and not necessarily those of Wellcome or the Department of Health. This study makes use of DECIPHER (https://www.deciphergenomics.org/), which is funded by the Wellcome Trust.

Declaration of interests

M.E.H. is a founder of, consultant to, director of, and holds shares in Congenica Ltd and is a consultant to the AZ Centre for

Genomics Research. H.V.F. is a Section Editor for genetics for UpToDate. All other authors declare no conflict of interest.

Received: October 8, 2020
Accepted: September 15, 2021
Published: October 8, 2021

Web resources

pysam: htslib interface for python, https://pysam.readthedocs.io/en/latest/

References

1. Collins, R.L., Brand, H., Karczewski, K.J., Zhao, X., Alföldi, J., Francioli, L.C., Khera, A.V., Lowther, C., Gauthier, L.D., Wang, H., et al.; Genome Aggregation Database Production Team; and Genome Aggregation Database Consortium (2020). A structural variation reference for medical and population genetics. Nature *581*, 444–451.
2. Zhao, X., Collins, R.L., Lee, W.-P., Weber, A.M., Jun, Y., Zhu, Q., Weisburd, B., Huang, Y., Audano, P.A., Wang, H., et al.; Human Genome Structural Variation Consortium (2021). Expectations and blind spots for structural variation detection from long-read assemblies and short-read genome sequencing technologies. Am. J. Hum. Genet. *108*, 919–928.
3. Gardner, E.J., Prigmore, E., Gallone, G., Danecek, P., Samocha, K.E., Handsaker, J., Gerety, S.S., Ironfield, H., Short, P.J., Sifrim, A., et al. (2019). Contribution of retrotransposition to developmental disorders. Nat. Commun. *10*, 4630.
4. Sanchis-Juan, A., Stephens, J., French, C.E., Gleadall, N., Mégy, K., Penkett, C., Shamardina, O., Stirrups, K., Delon, I., Dewhurst, E., et al. (2018). Complex structural variants in Mendelian disorders: identification and breakpoint resolution using short- and long-read genome sequencing. Genome Med. *10*, 95.
5. Torene, R.I., Galens, K., Liu, S., Arvai, K., Borroto, C., Scuffins, J., Zhang, Z., Friedman, B., Sroka, H., Heeley, J., et al. (2020). Mobile element insertion detection in 89,874 clinical exomes. Genet. Med. *22*, 974–978.
6. Kosugi, S., Momozawa, Y., Liu, X., Terao, C., Kubo, M., and Kamatani, Y. (2019). Comprehensive evaluation of structural variation detection algorithms for whole genome sequencing. Genome Biol. *20*, 117.
7. Kaplanis, J., Samocha, K.E., Wiel, L., Zhang, Z., Arvai, K.J., Eberhardt, R.Y., Gallone, G., Lelieveld, S.H., Martin, H.C., McRae, J.F., et al.; Deciphering Developmental Disorders Study (2020). Evidence for 28 genetic disorders discovered by combining healthcare and research data. Nature *586*, 757–762.
8. Schwarze, K., Buchanan, J., Taylor, J.C., and Wordsworth, S. (2018). Are whole-exome and whole-genome sequencing approaches cost-effective? A systematic review of the literature. Genet. Med. *20*, 1122–1130.
9. Fromer, M., Moran, J.L., Chambert, K., Banks, E., Bergen, S.E., Ruderfer, D.M., Handsaker, R.E., McCarroll, S.A., O'Donovan, M.C., Owen, M.J., et al. (2012). Discovery and statistical genotyping of copy-number variation from whole-exome sequencing depth. Am. J. Hum. Genet. *91*, 597–607.
10. Srivastava, S., Love-Nichols, J.A., Dies, K.A., Ledbetter, D.H., Martin, C.L., Chung, W.K., Firth, H.V., Frazier, T., Hansen, R.L., Prock, L., et al.; NDD Exome Scoping Review Work Group (2019). Meta-analysis and multidisciplinary consensus statement: exome sequencing is a first-tier clinical diagnostic test for individuals with neurodevelopmental disorders. Genet. Med. *21*, 2413–2421.
11. McKenna, A., Hanna, M., Banks, E., Sivachenko, A., Cibulskis, K., Kernytsky, A., Garimella, K., Altshuler, D., Gabriel, S., Daly, M., and DePristo, M.A. (2010). The Genome Analysis Toolkit: a MapReduce framework for analyzing next-generation DNA sequencing data. Genome Res. *20*, 1297–1303.
12. Short, P.J., McRae, J.F., Gallone, G., Sifrim, A., Won, H., Geschwind, D.H., Wright, C.F., Firth, H.V., FitzPatrick, D.R., Barrett, J.C., and Hurles, M.E. (2018). De novo mutations in regulatory elements in neurodevelopmental disorders. Nature *555*, 611–616.
13. Lord, J., Gallone, G., Short, P.J., McRae, J.F., Ironfield, H., Wynn, E.H., Gerety, S.S., He, L., Kerr, B., Johnson, D.S., et al.; Deciphering Developmental Disorders study (2019). Pathogenicity and selective constraint on variation near splice sites. Genome Res. *29*, 159–170.
14. Kaplanis, J., Akawi, N., Gallone, G., McRae, J.F., Prigmore, E., Wright, C.F., Fitzpatrick, D.R., Firth, H.V., Barrett, J.C., Hurles, M.E.; and Deciphering Developmental Disorders study (2019). Exome-wide assessment of the functional impact and pathogenicity of multinucleotide mutations. Genome Res. *29*, 1047–1056.
15. Liaw, A., and Wiener, M. (2002). Classification and Regression by randomForest. R News *2*, 18–22.
16. Li, H., and Durbin, R. (2010). Fast and accurate long-read alignment with Burrows-Wheeler transform. Bioinformatics *26*, 589–595.
17. Camacho, C., Coulouris, G., Avagyan, V., Ma, N., Papadopoulos, J., Bealer, K., and Madden, T.L. (2009). BLAST+: architecture and applications. BMC Bioinformatics *10*, 421.
18. Li, H., Handsaker, B., Wysoker, A., Fennell, T., Ruan, J., Homer, N., Marth, G., Abecasis, G., Durbin, R.; and 1000 Genome Project Data Processing Subgroup (2009). The Sequence Alignment/Map format and SAMtools. Bioinformatics *25*, 2078–2079.
19. Chen, X., Schulz-Trieglaff, O., Shaw, R., Barnes, B., Schlesinger, F., Källberg, M., Cox, A.J., Kruglyak, S., and Saunders, C.T. (2016). Manta: rapid detection of structural variants and indels for germline and cancer sequencing applications. Bioinformatics *32*, 1220–1222.
20. Zook, J.M., Catoe, D., McDaniel, J., Vang, L., Spies, N., Sidow, A., Weng, Z., Liu, Y., Mason, C.E., Alexander, N., et al. (2016). Extensive sequencing of seven human genomes to characterize benchmark reference materials. Sci. Data *3*, 160025.
21. Zook, J.M., Hansen, N.F., Olson, N.D., Chapman, L., Mullikin, J.C., Xiao, C., Sherry, S., Koren, S., Phillippy, A.M., Boutros, P.C., et al. (2020). A robust benchmark for detection of germline large deletions and insertions. Nat. Biotechnol. *38*, 1347–1355.
22. Thormann, A., Halachev, M., McLaren, W., Moore, D.J., Svinti, V., Campbell, A., Kerr, S.M., Tischkowitz, M., Hunt, S.E., Dunlop, M.G., et al. (2019). Flexible and scalable diagnostic filtering of genomic variants using G2P with Ensembl VEP. Nat. Commun. *10*, 2373.
23. Thorvaldsdóttir, H., Robinson, J.T., and Mesirov, J.P. (2013). Integrative Genomics Viewer (IGV): high-performance genomics data visualization and exploration. Brief. Bioinform. *14*, 178–192.
24. Karczewski, K.J., Francioli, L.C., Tiao, G., Cummings, B.B., Alföldi, J., Wang, Q., Collins, R.L., Laricchia, K.M., Ganna, A., Birnbaum, D.P., et al.; Genome Aggregation Database Consortium

(2020). The mutational constraint spectrum quantified from variation in 141,456 humans. Nature *581*, 434–443.

25. Wright, C.F., McRae, J.F., Clayton, S., Gallone, G., Aitken, S., FitzGerald, T.W., Jones, P., Prigmore, E., Rajan, D., Lord, J., et al.; DDD Study (2018). Making new genetic diagnoses with old data: iterative reanalysis and reporting from genome-wide data in 1,133 families with developmental disorders. Genet. Med. *20*, 1216–1223.
26. Amir, R.E., Van den Veyver, I.B., Wan, M., Tran, C.Q., Francke, U., and Zoghbi, H.Y. (1999). Rett syndrome is caused by mutations in X-linked MECP2, encoding methyl-CpG-binding protein 2. Nat. Genet. *23*, 185–188.
27. Lek, M., Karczewski, K.J., Minikel, E.V., Samocha, K.E., Banks, E., Fennell, T., O'Donnell-Luria, A.H., Ware, J.S., Hill, A.J., Cummings, B.B., et al.; Exome Aggregation Consortium (2016). Analysis of protein-coding genetic variation in 60,706 humans. Nature *536*, 285–291.
28. Neul, J.L., Kaufmann, W.E., Glaze, D.G., Christodoulou, J., Clarke, A.J., Bahi-Buisson, N., Leonard, H., Bailey, M.E.S., Schanen, N.C., Zappella, M., et al.; RettSearch Consortium (2010). Rett syndrome: revised diagnostic criteria and nomenclature. Ann. Neurol. *68*, 944–950.
29. Guy, J., Alexander-Howden, B., FitzPatrick, L., DeSousa, D., Koerner, M.V., Selfridge, J., and Bird, A. (2018). A mutation-led search for novel functional domains in MeCP2. Hum. Mol. Genet. *27*, 2531–2545.
30. Watson, P., Black, G., Ramsden, S., Barrow, M., Super, M., Kerr, B., and Clayton-Smith, J. (2001). Angelman syndrome phenotype associated with mutations in MECP2, a gene encoding a methyl CpG binding protein. J. Med. Genet. *38*, 224–228.
31. Bebbington, A., Percy, A., Christodoulou, J., Ravine, D., Ho, G., Jacoby, P., Anderson, A., Pineda, M., Ben Zeev, B., Bahi-Buisson, N., et al. (2010). Updating the profile of C-terminal MECP2 deletions in Rett syndrome. J. Med. Genet. *47*, 242–248.
32. Wright, C.F., Fitzgerald, T.W., Jones, W.D., Clayton, S., McRae, J.F., van Kogelenberg, M., King, D.A., Ambridge, K., Barrett, D.M., Bayzetinova, T., et al.; DDD study (2015). Genetic diagnosis of developmental disorders in the DDD study: a scalable analysis of genome-wide research data. Lancet *385*, 1305–1314.
33. Ye, K., Schulz, M.H., Long, Q., Apweiler, R., and Ning, Z. (2009). Pindel: a pattern growth approach to detect break points of large deletions and medium sized insertions from paired-end short reads. Bioinformatics *25*, 2865–2871.
34. Krishnaraj, R., Ho, G., and Christodoulou, J. (2017). RettBASE: Rett syndrome database update. Hum. Mutat. *38*, 922–931.
35. Huppke, P., Maier, E.M., Warnke, A., Brendel, C., Laccone, F., and Gärtner, J. (2006). Very mild cases of Rett syndrome with skewed X inactivation. J. Med. Genet. *43*, 814–816.
36. Gardner, E.J., Lam, V.K., Harris, D.N., Chuang, N.T., Scott, E.C., Pittard, W.S., Mills, R.E., Devine, S.E.; and 1000 Genomes Project Consortium (2017). The Mobile Element Locator Tool (MELT): population-scale mobile element discovery and biology. Genome Res. *27*, 1916–1929.

The American Journal of Human Genetics, Volume *108*

Supplemental information

Detecting cryptic clinically relevant structural variation in exome-sequencing data increases diagnostic yield for developmental disorders

Eugene J. Gardner, Alejandro Sifrim, Sarah J. Lindsay, Elena Prigmore, Diana Rajan, Petr Danecek, Giuseppe Gallone, Ruth Y. Eberhardt, Hilary C. Martin, Caroline F. Wright, David R. FitzPatrick, Helen V. Firth, and Matthew E. Hurles

Supplemental Figures

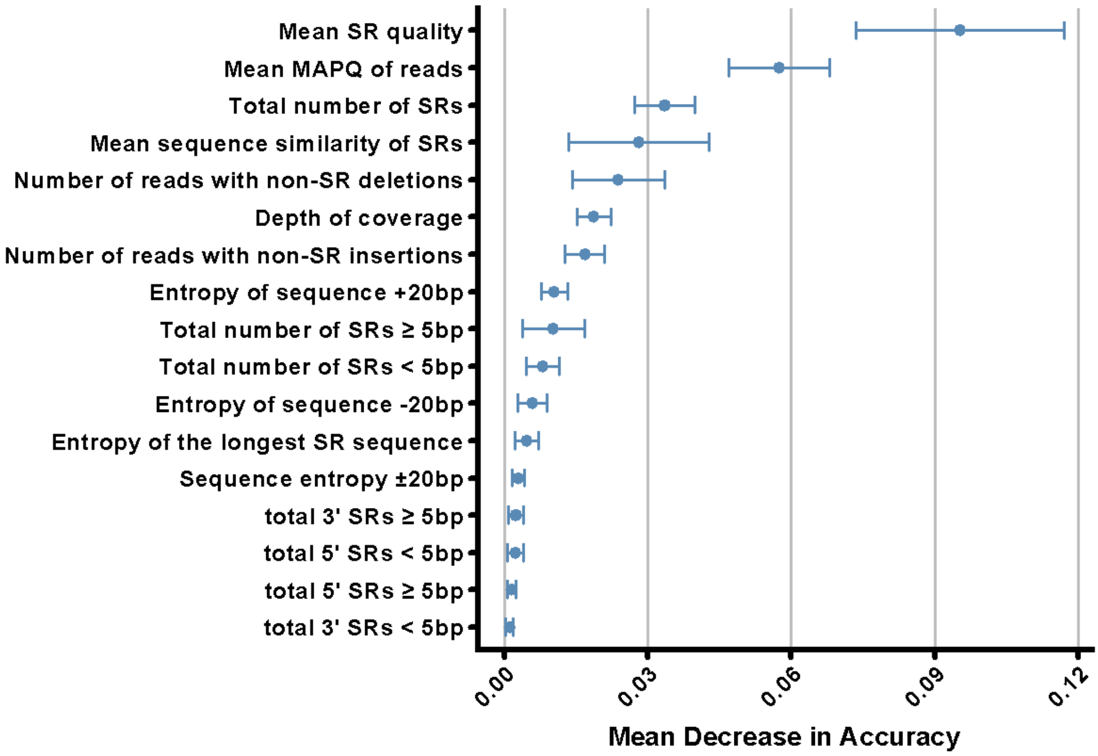

Figure S1: Feature importance from the InDelible random forest.

Shown is the mean decrease in accuracy given by random forest for each of the 17 features we used to estimate the probability of a split read cluster being a true positive variant. Error bars represent standard deviation of mean decrease in accuracy for 10 cross-validated random forests at k = 75 (Figure S4) for the final forest generated by our active learning model (see Supplemental Methods). Features which quantify the number of reads with clipped sequence length less than/greater than 5bp (e.g. Total number of SRs ≥ 5bp) represents the default setting of InDelible and can be adjusted by the end-user during random forest training. Sequence entropy for all categories is calculated as in Schmitt and Herzel[1]. Depth of coverage is calculated using the ‘genomecov’ module included as part of bedtools[2].

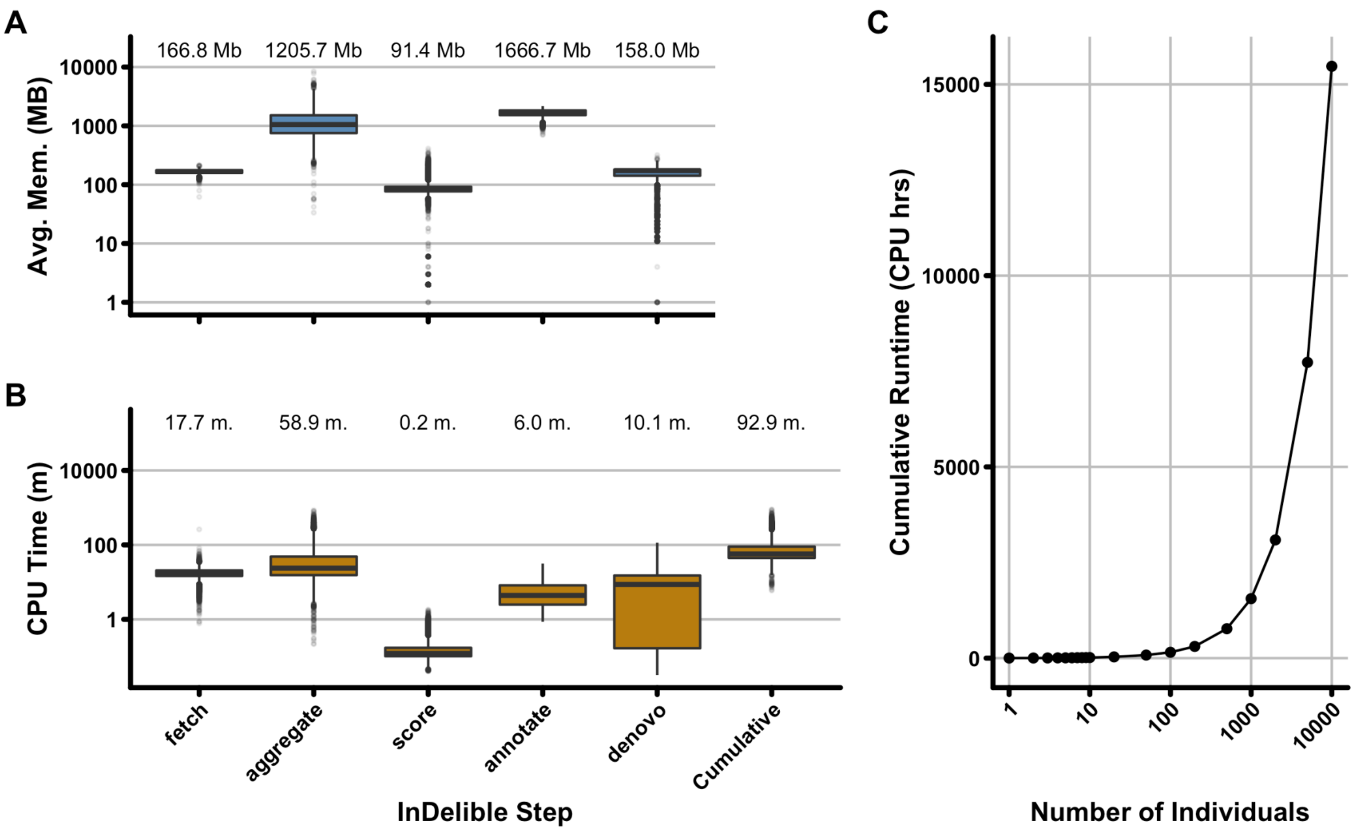

Figure S2: Benchmarks of InDelible.

Using real data from the DDD study, we benchmarked the InDelible algorithm. Average memory use in megabytes (**A**) and CPU time in minutes (**B**) across all 13,438 DDD probands for the five steps of the InDelible SV discovery pipeline which run on individual samples (i.e. other than "Database"; Main Text Figure 1). Using the CPU times in (**B**), we extrapolated runtimes for studies of various sizes by randomly sampling the number of individuals shown on the x-axis (**C**).

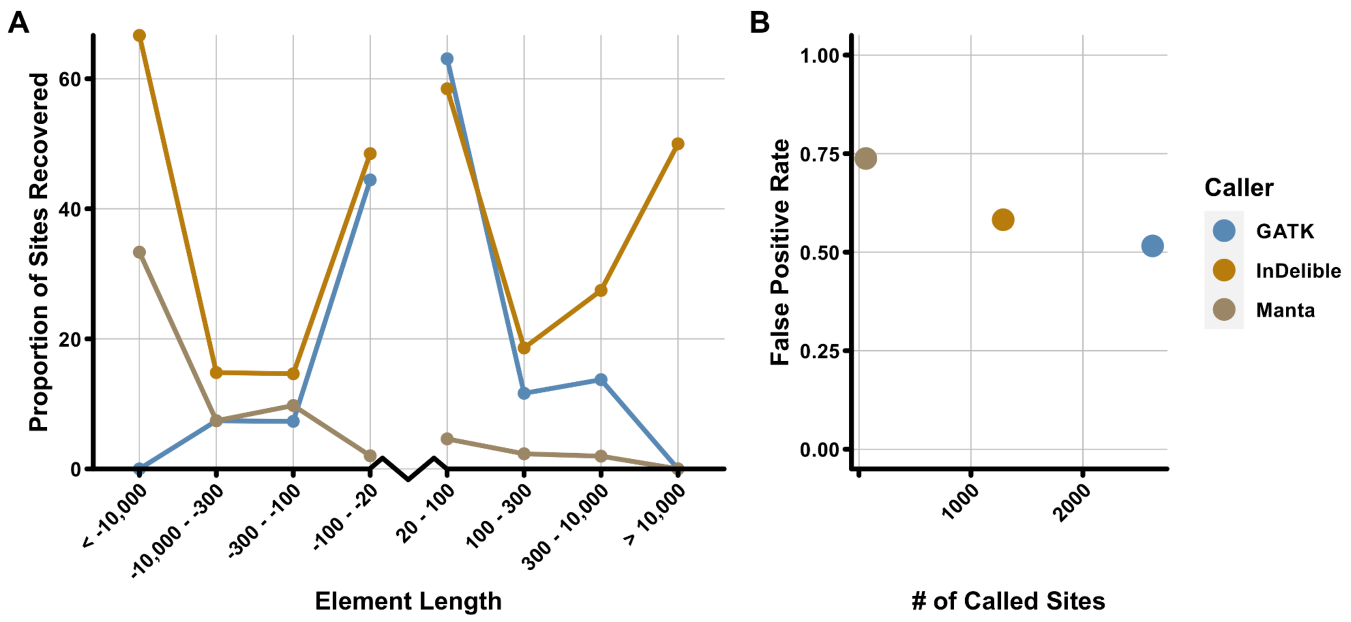

Figure S3: InDelible benchmarks across the allele frequency spectrum.

To benchmark InDelible, we called variants using InDelible, GATK, and Manta in the genome of an Ashkanazi Jewish individual characterised for both small InDels and large structural variants by the Genome in a Bottle (GIAB) consortium (Supplemental Methods)[3,4]. (**A**) Recall rate of all three callers for deletions (left side of plot) and insertions/duplications (right side of plot). Each point represents the proportion of variants in the size bin depicted on the x-axis. Size-ranges for deletions and insertions/duplications are right-open and left-open, respectively. Variants with an absolute size ≤20 base pairs were excluded from this analysis. Despite GATK generally being designed to identify variants <100bps in length, for a limited set of variants GATK was able to ascertain at least one breakpoint for some larger variants (i.e. > 100bps), albeit with incorrect size estimates. (**B**) False positive rate for all three callers as a function of total number of sites identified by each caller. We note that these experiments likely drastically overstate the FP rates of all callers – GIAB gold-standard calls were based on a merge of several different technologies and variants that are readily identifiable with long-read sequencing (e.g. Pacific Biosciences sequencing) may be difficult to identify with short-read based approaches which query ES data.

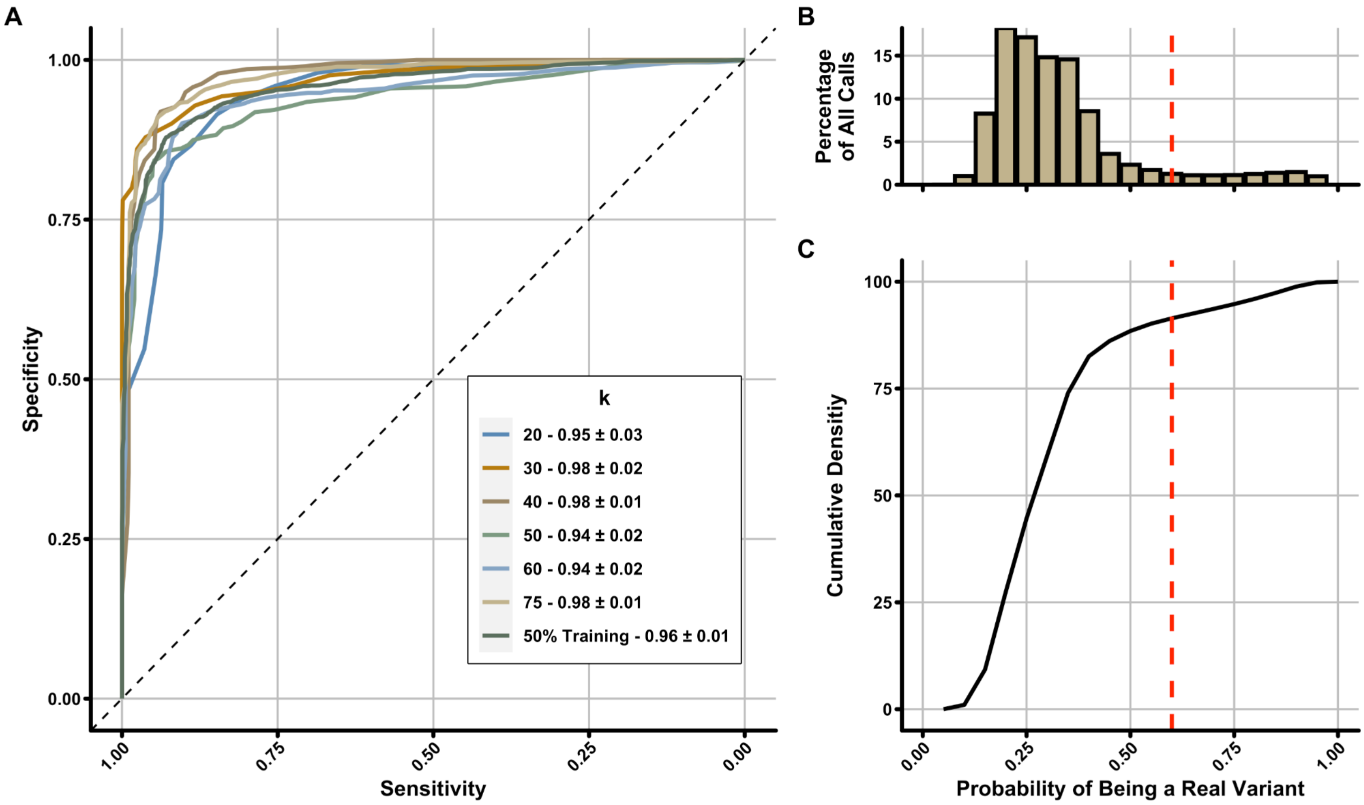

Figure S4: Filtering split read clusters in InDelible with active learning.

To estimate the probability of a variant being real, we utilized an active learning approach (see Supplemental Methods). (**A**) Cross validated ROC curves at various inputs of *k* as well as for a traditional machine learning model where data was split into 50% training and test data without active learning. (**B**) Total number of original redundant split read clusters in 0.05 p-value bins. By default, InDelible filters all split read clusters with a probability of being a true variant <0.6 (red dashed line). (**C**) Cumulative density plot of the redundant clusters from (**B**).

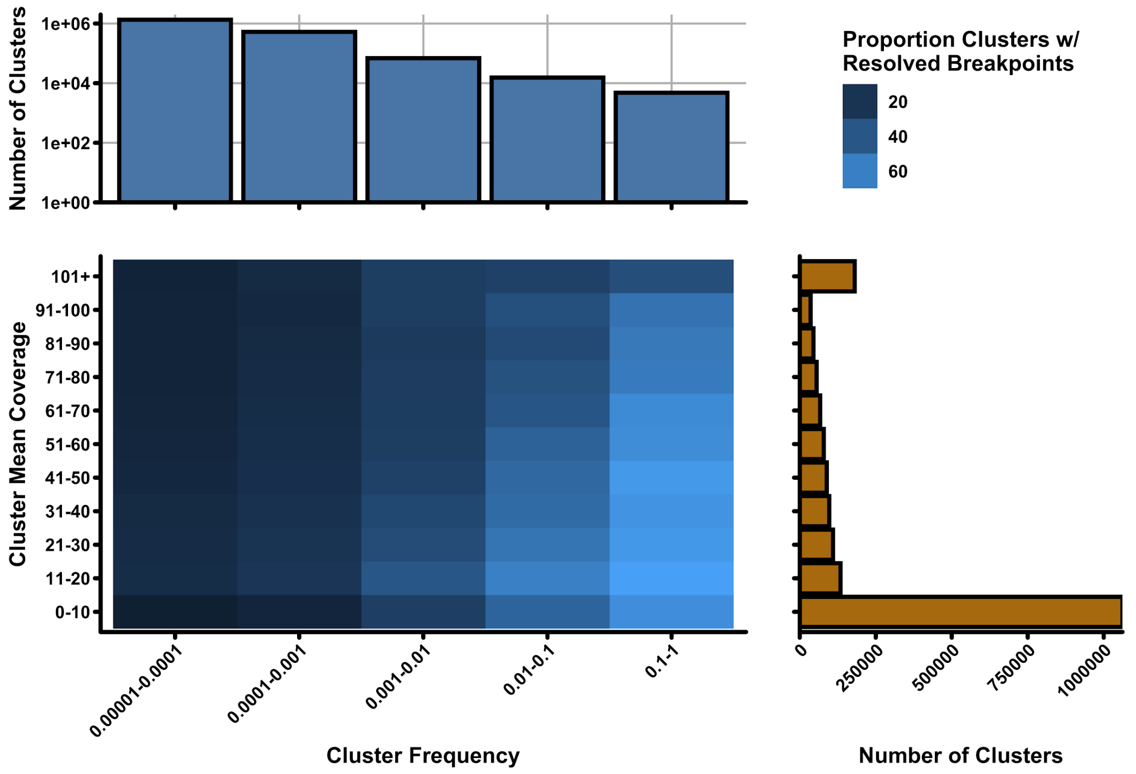

Figure S5: Breakpoint resolution of InDelible as a factor of allele frequency and coverage.

The lower-left plot represents the proportion of sites within each bin on the X (cluster frequency – proportion of individuals a given site has been identified in) and Y (cluster mean coverage – mean sequencing coverage within individuals with a given cluster) axes which have resolved variant type and/or breakpoints. The lighter the shade of blue, the higher proportion of sites within that bin that have resolved breakpoints. Marginal histograms represent the total number of clusters within each bin on the X and Y-axes.

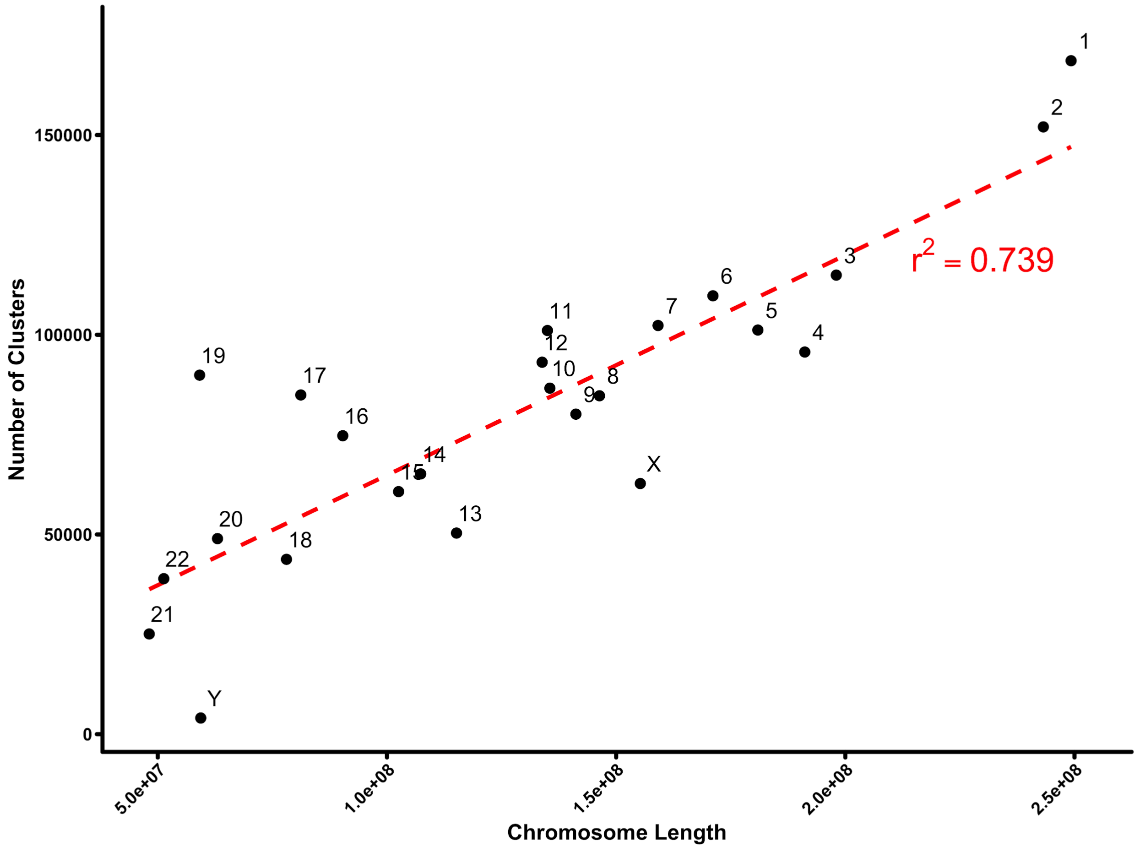

Figure S6: Number of identified breakpoints per-chromosome.

Total number of breakpoint clusters identified on the 22 autosomes and 2 allosomes in the Deciphering Developmental Disorders study by InDelible. The r^2 value represents the correlation coefficient for Number of Clusters ~ Chromosome Length.

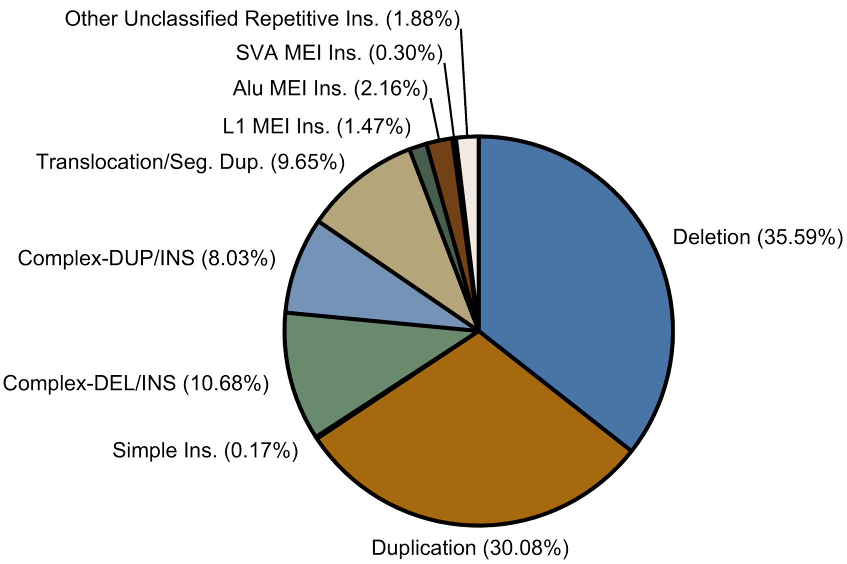

Figure S7: Variant types identified by InDelible.

InDelible identifies a wide range of genetic rearrangement classes using a re-alignment of split read sequences to the reference genome. Each slice of the pie represents the proportion of breakpoints exhibiting the annotated structure among sites that have both 5'/3' breakpoints resolved or align to a known human repeat element (total n = 199,932 breakpoints). Translocations and segmental duplications (Translocation/Seg. Dup. above) are grouped together as discerning between these variant types is difficult with available sequencing data.

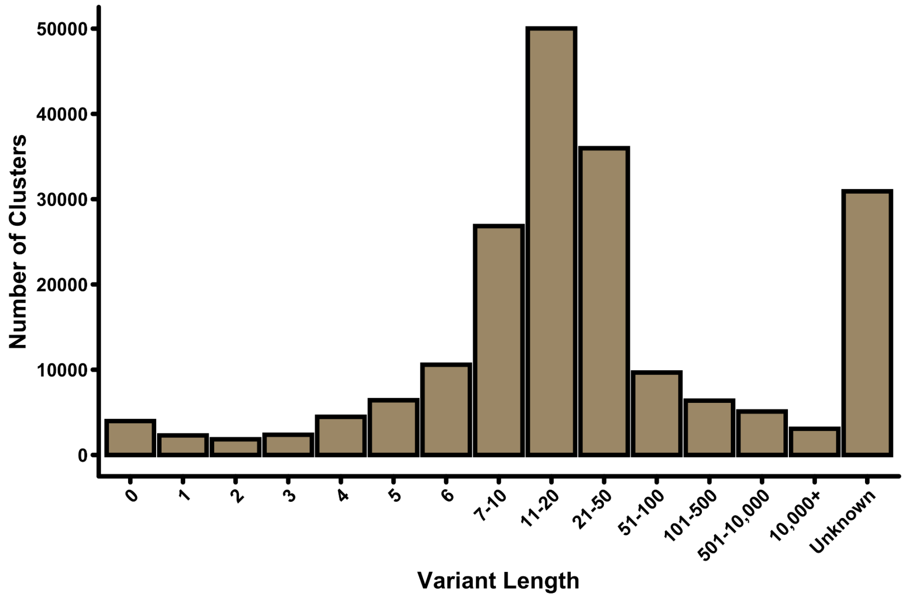

Figure S8: Variants by resolved length.

Variants InDelible is able to generate length estimates for mostly fall between 10-100 base pairs in length. Variants with a size of “0” are balanced rearrangements. Variants with “Unknown” length are either translocations, segmental duplications, or mobile element insertions. InDelible does not attempt to generate length estimates for these three variant classes.

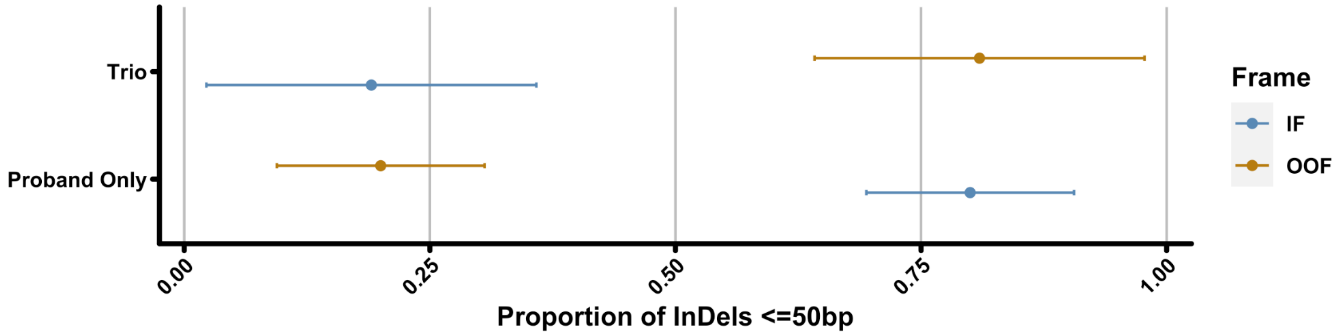

Figure S9: Proportion of in-frame versus out-of-frame insertions for trio versus non-trio data.

Shown is the proportion of variants ≤50bp which are in-frame (blue) or out-of-frame (orange) separated by whether the variant was called in a proband sequenced with/without parental samples.

Supplemental Tables

Library Name	Version	Website	Citation (If Applicable)
Cython	0.29.13	https://cython.org/	Behnel, et al.[5]
PyYAML	5.1.2	https://pyyaml.org/	n.a.
Biopython	1.74	https://biopython.org/	Cock, et al.[6]
Intervaltree	3.0.2	https://github.com/chaimleib/intervaltree	n.a.
numpy	1.17.2	https://numpy.org/	Harris, et al.[7]
pandas	0.25.1	https://pandas.pydata.org/	McKinney[8]
pyfaidx	0.5.5.2	https://pythonhosted.org/pyfaidx/	Shirley, et al.[9]
pysam	0.15.3	https://github.com/pysam-developers/pysam	n.a.
scikit-learn	0.21.3	https://scikit-learn.org/stable/	Pedregosa, et al.[10]
scipy	1.3.1	https://www.scipy.org/	Virtanen, et al.[11]

Table S1: Python libraries used by InDelible.

“Version” reflects the version number of the named package used during software development and for all analyses performed in this manuscript.

Supplemental Methods

Overview of the InDelible Algorithm

InDelible is open source software licensed under GPLv3.0 and developed in version 3.7 of the Python programming language. InDelible is freely available for academic use and detailed installation and usage instructions are available at GitHub (see main text Data and Code Availability). While all results in this manuscript use the version of InDelible built on Python3.7, InDelible has also been tested for backwards compatibility with Python2.7. InDelible additionally makes use of several open source libraries, all of which are listed in Table S1, and supports both CRAM and BAM input aligned with the bwa mem or aln algorithm[12].

A typical InDelible workflow proceeds in the steps outlined in main text Figure 1. Here we provide detailed descriptions of each of these steps:

- Fetch: Iterates through all reads in the provided sequence file and identifies those which have at least one end marked as clipped (i.e. with at least one "S" tag in the alignment cigar string) by bwa mem/aln[12]. Reads which, based on configurable user input, have a high map quality, split read length, and base quality are retained and provided as output for the next step.
- Aggregate: Reads which pass initial filtering in "Fetch" are then clustered based on chromosome and position. Clusters with fewer than a user-provided number of reads (default <3 reads), are discarded at this step. At each cluster, InDelible calculates features (Figure S1) that will then be used as input to random forest (Figure S4). At this step, InDelible also quantifies the proportion of reads at each cluster which have both the 5' and 3' end marked as split for later quality control (see "Detailed InDelible Variant Calling Protocol" below).
- Score: Each split read cluster is then annotated with the probability of being a true variant, regardless of clinical significance or *de novo* status, based on a 500-tree forest trained on manually curated and visualized truth data from DDD trios (see below)[10]. By default, clusters which have a probability <0.6 of being true sites are filtered at this step (Figure S4).

- Database: Calculates allele frequency, longest alternate sequence, and SV class (i.e. deletion, duplication, insertion, etc.) from a provided set of files generated by the "Score" command. Calculation of frequency is done by simple overlap across all provided files, and does not consider nearby loci. For all merged loci, InDelible then attempts to find the longest split-read sequence across all merged individuals by identifying the longest sequence with at least 60% homology to all other sequences using the 'difflib' package for base Python. This sequence is then aligned to a set of curated human repeat sequences with the BLAST implementation included as part of biopython[6,13] and queried against the provided reference genome using bwa mem[12]. For bwa mem alignment, sequences are also paired with a synthetic read extracted from up or downstream reference sequence to anchor the event. SV type, alternate sequence, breakpoints, and variant length are then extracted from bwa alignments. Only bwa alignments with mapping quality of both reads > 0, alignment orientation of both reads in the sense strand, and with the "reference" read anchored at the expected position are retained. While alignment orientation other than in the sense direction can be expected for a small number of variant types such as inversions, we opted to use this filtering regime to remove false positives. This filtering approach does not affect variants with at least one breakpoint directly impacting the coding sequence of genes included in the DDG2P target panel. The database step can also take a collection of priors; InDelible comes pre-packaged with a database generated from all probands analysed in this study.
- Annotate: Split read clusters are annotated with gene intersect to all ENSEMBL genes[14], disruption of coding sequence, and constraint based on the probability of being haploinsufficient[15]. A second gene list can also be provided which represents genes the end-user is specifically interested in (in the case of this manuscript, monoallelic Developmental Disorders Genotype-to-Phenotype database (DDG2P) genes[16]). Finally, the proportion of samples which also contain this cluster (i.e. allele frequency) is determined for each locus from the user-provided allele frequency database. All inputs at this stage can be modified by the end user to better fit specific project requirements.
- denovo: When provided, InDelible will query parental sequencing files for presence/absence of each split read cluster. Following assessment, clusters are then filtered if the total number of supporting split reads in either parental sample is too high (default >3 reads) and at sufficient coverage (default

≥9x). When parental samples are not available, "denovo" reformats the output from "Annotate" to make it consistent with output from probands sequenced as part of a trio; columns requiring parental information are provided as "NA".

Two additional commands are also provided with InDelible which add additional functionality depending on the needs of the user:

- Complete: Runs all of the above commands in succession for one sample.
- Train: Trains the random forest which InDelible uses to determine likelihood of being a true variant for each split read cluster. This method uses the random forest implementation provided as part of the Python package scikit-learn[10] combined with active learning. Active learning uses an iterative approach whereby a test and training set of size k are constructed from a pool of n variants of known truth with equal class balance, with all remaining variants (i.e. $n - k * 2$) placed into a validation set – the goal of active learning is to focus model training on the categorization of variants that are most difficult to classify. The random forest is then constructed with the training set and accuracy of the model determined via prediction on the test set using several covariates calculated during the 'Aggregate' step (Figure S1). k sites are then moved from the validation set to the training set based on their inability to be classified compared to other variants ($|P(TP) - P(TN)|$). The random forest is then retrained using the new training set until the change in prediction accuracy of the test set between rounds is less than 1%. Random forest settings as implemented by scikit-learn are set to default except the number of computed decision trees, which is set to 500. To avoid issues with class balance, the Train command randomly samples the same number of false sites as true sites. InDelible includes training data comprising variants ascertained from the DDD study and the resulting random forest model, but can be retrained with alternative data.

As part of the InDelible distribution, when possible, we have provided genomic reference files such as gene models, DDG2P genes included in this study, and repeat sequences. Instructions to download files that are not provided (e.g. the human reference) are provided as part of our github repository. The output of the "Database" and "Train" commands generated with DDD data are also provided (see Data and Code

Availability in the main text), but in most cases it is highly recommended to regenerate these files with data specific to the current project.

We have also generated a Docker image containing the version of InDelible used to analyse the DDD data included in this manuscript. Functionality is identical to that described above. Please see the Data and Code Availability section of the main text for more information on acquisition, installation, and usage of the Dockerised version of InDelible.

DDD Study Recruitment and Sequencing

A total of 13,451 individuals were recruited from 24 regional genetics services throughout the United Kingdom and Republic of Ireland as previously described[17]. Sequencing of families and alignment to the human reference genome (GRCh37) with bwa[12] was performed as previously described[17] but is repeated here in brief. Genomic DNA from all samples (probands and recruited parents, if available) recruited to DDD were fragmented to an average size of 150bp and used to create Illumina PCR-amplified paired-end libraries. Libraries were then hybridized to one of two SureSelect RNA baits (v3 or v5), captured, amplified and submitted for 75bp paired-end sequencing on an Illumina HiSeq following manufacturer instructions. All samples were sequenced to a mean depth of 90x across primary bait capture regions. Following sequencing, all samples were aligned with either bwa aln or mem[12] to the 1000 Genomes Project phase 2 human reference (vers. hs37d5) and processed with IndelRealigner and Base Quality Score Recalibration (BQSR) available as part of the GATK resource bundle (version 2.2).

InDelible Variant Calling Protocol

To ascertain the variants reported in this manuscript, we applied InDelible to all 13,451 recruited probands, but excluded 13 probands from further analysis due to excessive runtime, leaving 13,438 probands. This includes probands sequenced with both parents (trios, n = 9,848) or with one or both parents absent (non-trios, n = 3,590). As this was the first dataset analyzed with InDelible, we ran all steps while also training the random forest (Figure 1).

All probands were first run through the “Fetch” and “Aggregate” steps with default settings to identify 353,313,108 redundant split read clusters. Next, to train our random forest to perform split read cluster

quality control, we randomly selected 2,000 non-redundant sites across all probands from the output of the aggregate step. We then visually inspected all 2,000 sites using the Integrative Genomics Viewer[18] to build a labelled truth set of variants for training. These 2,000 manually curated sites were then provided as input to the "Train" subcommand of InDelible at various test and training sizes (k). We ultimately decided on a probability (p) of being a true variant $p > 0.6$ at $k = 75$ as a reasonable value for filtering following cross-validation (Figure S4). We then used the trained random forest to score all sites identified with the probability of being a true variant with the InDelible "Score" command. Training data used for this study is available as part of the GitHub repository provided in the Data and Code Availability section of the main text.

We next provided all output files of the "Score" command for all probands as input to the "Database" command with default settings to build the allele frequency database required as input to the "Annotate" command. All probands and split read clusters were subsequently processed with the "Annotate" and "denovo" commands with default settings. Following initial calling, we performed additional quality control to generate our final set of putatively clinically relevant variants. Split read clusters were retained at this step based on the following criteria:

1. Breakpoint frequency < $4x10^{-4}$
2. Average MAPQ ≥ 20
3. Number of split reads ≥ 5
4. Proportion of split reads as a factor of coverage ≥ 0.1 (i.e. sr_total / coverage)
5. Affecting coding sequence (here defined as exons ±10bp)
6. Intersected a gene with a known monoallelic, X-linked, or hemizygous mechanism with a loss of function or dominant negative consequence based off of the DDG2P database[19]
7. < 2 split reads in either the maternal or paternal sample, if available
8. < 50% of reads with both ends split (i.e. both 5' and 3' ends with an "S" tag in the cigar string) AND
    a. ≤ 10% of reads with both ends split OR
    b. > 10% of reads with both ends split while also having a valid bwa alignment (see "Database" step)

A script which performs this filtering is included at the InDelible GitHub repository (see main text Data and Code Availability). Calling and subsequent filtering left a remainder of 354 split read clusters. Split read clusters identified at the same locus within the same proband were subsequently manually merged (marked as "OTHERSIDE" in Table S2), leaving a remainder of 260 variants, with the 5' breakpoint retained for final reporting.

Via visual inspection of read alignments[18], we next determined whether variants were likely to be real in the proband and inherited from a parent where possible (Figure 2A). Variants with gnomAD non-Finnish European allele frequency ≥ $1x10^{-4}$ based on Karczewski et al.[20] (variants ≤ 50bp) or Collins et al.[21] (variants > 50bp) were then filtered out from further analysis (Figure 2A, Table S2). This filtering approach based on reference datasets is imperfect – as the variant size range that InDelible detects is under-represented, some variants may be common in the population but may not be represented in such reference datasets. To determine the sensitivity of InDelible for DDD variants previously reported as potentially pathogenic, InDelible variants were intersected with previously reported DDD variants[19] and with CNVs called from read-depth analysis of DDD ES data (Table S2). Previously known variants, false negative/positive variants, variants with high DDD/gnomAD allele frequency, variants that do not actually intersect a known DD gene, and variants located in regions difficult to interpret clinically (e.g. intron or 5'/3' UTR) are annotated as such in Table S2.

To identify a set of rare inherited variants that could plausibly be associated with DDD study participant phenotype, we repeated our above filtering as for *de novo* variants except we restricted to variants found only in a single proband (e.g. singletons) without filtering for parental split read support. This approach identified a total of 211 breakpoints which, after collapsing identical loci as above, left a total of 145 variants for downstream analysis. We next excluded variants based on likely association with DDD study participant phenotype by removing:

- In-frame InDels inherited from an unaffected parent.
- Primarily non-coding variants.
- Variants found in individuals with a more plausible variant already reported.
- Variant types of uncertain consequence such as processed pseudogenes and duplications which partially overlap coding sequence.

- Presence in any control individuals in the gnomAD database.
- Likely *de novo* variants already ascertained by InDelible and/or other approaches

Note that many variants fulfill multiple of the above criteria. This filtering left a total of 17 variants, of which 7 were already identified by an approach other than InDelible and returned to referring clinicians. The remaining 10 variants were annotated for contribution to DDD study participant phenotype, returned to referring clinicians where relevant, and provided in Table S4.

Benchmarking InDelible Runtime and Memory

To benchmark InDelible, we collected usage statistics provided by the standard output of the Platform Load Sharing Facility (LSF) at the Wellcome Sanger Institute. Metrics were generated during the course of processing the 13,438 DDD individuals through the standard InDelible SV discovery pipeline. The compute cluster used consists of 105 nodes with 32 8-core 2400Ghz CPU AMD Opteron Processors with 256Gb of memory each. On average, InDelible took 92.9 CPU minutes and a maximum of 1.7Gb of memory to run one sample with mean exome-wide coverage of 90x from aligned CRAM file to reporting of candidate diagnostic variants (Figure S2). Considering that most users would likely be utilizing InDelible to analyse multi-sample datasets, we also calculated extrapolated runtimes via down-sampling of our own DDD runtimes for datasets composed of between 1-10,000 individuals (Figure S2). To generate the extrapolated curve seen in Figure S2C, we randomly sampled individuals 100 times at the sample size indicated on the x-axis to generate an average expected runtime.

PCR Validation

To validate all 54 variants returned to clinicians via the DECIPHER platform, we used PCR. For small variants (≤50bp) we extracted the surrounding 300bp and used Primer3[22] with GC clamp turned on to automatically design an initial set of primers. Following initial design, we confirmed specificity with UCSC *in silico* PCR. For larger variants, we manually designed primers 5' and 3' of the predicted variant breakpoint and likewise confirmed specificity with UCSC *in silico* PCR. PCR was carried out using REDAccuTaq® LA DNA Polymerase (Sigma); 80ng of genomic DNA extracted from blood or saliva was amplified in the presence of 0.4 µM of each primer and 1 unit of REDAccuTaq. We used the following cycling conditions:

30s at 98°C followed by 6 cycles of (15s at 94°C, 45s at 60°, and 60s at 68°C) and 24 cycles of (15s at 94°C, 45s at 55°, and 60s at 68°C). For longer variants (i.e. ≥1Kbp) elongation time was changed from 45s to 480s. A final elongation step was carried out for 30m at 68°C. PCR products were visualized on a 2% or 4% agarose gel for short and long products, respectively. Following PCR, all reactions were PCR purified using a standard exosap protocol and submitted for capillary sequencing. Following sequencing, traces were examined for quality and BLAT and manual inspection used to check for presence of the predicted variant. For 28 variants we were unable to obtain conclusive validation results due to too little DNA for PCR (n = 3), failed capillary sequencing (n = 8), failed primer design (n = 6), or failed PCR (n = 11). We were unable to allocate resources during the COVID-19 pandemic to validate 3 further variants prior to returning them to referring clinicians.

Analysis of *MECP2* Carrier Phenotypes

For proband phenotypes reported in Figure 3B, we first collated all HPO terms as reported by the referring clinician for all probands with an InDelible-ascertained *MECP2* variant. We then condensed these terms into 19 super-HPO groups which group related HPO terms into a more broad phenotype (e.g. the terms long toe and long fingers condense into the digital anomalies super-HPO group). Original HPO terms for all probands and the super-HPO terms to which they were assigned are available in Table S3.

Proportion of PTVs Called By InDelible

To determine the proportion of PTVs within a dataset attributable to InDelible as shown in Figure 4, we queried variants identified in DDD probands sequenced as a trio from three different sources: (i) GATK called SNVs and short InDels ≤100bp from DECIPHER for DDD study participants (n = 1,140 variants)[23], (ii) XHMM called CNVs from CNV data generated for DDD probands (unpublished; n = 128 deletions), and (iii) MELT-ascertained[24] MEIs from Gardner et al.[25] that were plausibly associated with participant symptoms (n = 4 MEIs). For all variant types, we retained only *de novo* variants which overlapped the same set of DD genes used for InDelible ascertainment. For InDelible, we considered the 56 *de novo* variants reported in Table S2, regardless of novelty. InDelible Variants were then matched to each of the three other callsets,

first by exact coordinate/sequence match and then by subsequent manual confirmation. Variants queried for the purpose of this exercise were also used to catalogue previously reported *MECP2* variants.

Benchmarking InDelible using Genome in a Bottle Consortium Data

To benchmark InDelible against GATK and Manta, we downloaded ES data for sample HG002 (Ashkenazi proband sample) provided by the GIAB Consortium from the NCBI FTP site (https://github.com/genome-in-a-bottle/giab_data_indexes). We then acquired variant benchmarks for small variants from Zook et al. (2016)[3] and structural variants from Zook et al. (2020)[4] and limited each to variants with a REF/ALT size difference ≥1bp (i.e. InDels) and at least one breakpoint within padded (i.e. ±100bps) exome bait regions used for original whole exome sequencing (SureSelect coordinate files acquired from Agilent). We then ascertained variants from HG002 using InDelible, GATK, and Manta. For InDelible, we used the “complete” pipeline with default settings. Filtering was performed identically to that outlined for *de novo* variant discovery above, except we did not filter variants with high allele frequency, located outside coding sequence, or outside of known monoallelic DDG2P genes. For GATK we first ran HaplotypeCaller to generate a gVCF and then ran GenotypeGVCFs on the resulting output with default settings to generate a final output of genotyped sites. Default settings were used for GATK except we provided the SureSelect bait regions outlined above during the HaplotypeCaller step. We then used bcftools[26] to filter sites with GQ < 20 and DP < 7 to generate a final list of sites for benchmarking. To identify structural variant breakpoints with Manta, we first ran configManta.py with default settings other than providing ES baits with `--callRegions` and setting the `--exome` flag to generate a Manta workflow file. We then ran the resulting `runWorkflow.py` command.

We then converted all three callsets to bed format and used a custom python script to ask if any variant from HG002 as ascertained by the GIAB consortium was found within 100bps of a variant called by any of the three callers to calculate recall rate. All variants/breakpoints that were not within 100bps of a true variant were coded as false positives (Figure S3). To calculate recall relative to InDelible, we quantified the number of variants >20bps in length recalled by InDelible, GATK, and Manta separately for deletions and insertions/duplications. We then divided calculated values for GATK and Manta by the value for InDelible as presented in the main text.

Supplemental References

1. Schmitt, A.O., and Herzel, H. (1997). Estimating the entropy of DNA sequences. J. Theor. Biol. *188*, 369–377.

2. Quinlan, A.R. (2014). BEDTools: The Swiss-Army Tool for Genome Feature Analysis. Current Protocols in Bioinformatics *47*,.

3. Zook, J.M., Catoe, D., McDaniel, J., Vang, L., Spies, N., Sidow, A., Weng, Z., Liu, Y., Mason, C.E., Alexander, N., et al. (2016). Extensive sequencing of seven human genomes to characterize benchmark reference materials. Sci Data *3*, 160025.

4. Zook, J.M., Hansen, N.F., Olson, N.D., Chapman, L., Mullikin, J.C., Xiao, C., Sherry, S., Koren, S., Phillippy, A.M., Boutros, P.C., et al. (2020). A robust benchmark for detection of germline large deletions and insertions. Nat. Biotechnol. *38*, 1347–1355.

5. Behnel, S., Bradshaw, R., Citro, C., Dalcin, L., Seljebotn, D.S., and Smith, K. (2011). Cython: The Best of Both Worlds. Computing in Science & Engineering *13*, 31–39.

6. Cock, P.J.A., Antao, T., Chang, J.T., Chapman, B.A., Cox, C.J., Dalke, A., Friedberg, I., Hamelryck, T., Kauff, F., Wilczynski, B., et al. (2009). Biopython: freely available Python tools for computational molecular biology and bioinformatics. Bioinformatics *25*, 1422–1423.

7. Harris, C.R., Millman, K.J., van der Walt, S.J., Gommers, R., Virtanen, P., Cournapeau, D., Wieser, E., Taylor, J., Berg, S., Smith, N.J., et al. (2020). Array programming with NumPy. Nature *585*, 357–362.

8. McKinney, W. (2010). Data Structures for Statistical Computing in Python. Proceedings of the 9th Python in Science Conference.

9. Shirley, M.D., Ma, Z., Pedersen, B.S., and Wheelan, S.J. Efficient “pythonic” access to FASTA files using pyfaidx.

10. Pedregosa, F., Varoquaux, G., Gramfort, A., Michel, V., Thirion, B., Grisel, O., Blondel, M., Prettenhofer, P., Weiss, R., Dubourg, V., et al. (2011). Scikit-learn: Machine Learning in Python. J. Mach. Learn. Res. *12*, 2825–2830.

11. Virtanen, P., Gommers, R., Oliphant, T.E., Haberland, M., Reddy, T., Cournapeau, D., Burovski, E., Peterson, P., Weckesser, W., Bright, J., et al. (2020). SciPy 1.0: fundamental algorithms for scientific computing in Python. Nat. Methods *17*, 261–272.

12. Li, H., and Durbin, R. (2010). Fast and accurate long-read alignment with Burrows-Wheeler transform. Bioinformatics *26*, 589–595.

13. Camacho, C., Coulouris, G., Avagyan, V., Ma, N., Papadopoulos, J., Bealer, K., and Madden, T.L. (2009). BLAST+: architecture and applications. BMC Bioinformatics *10*, 421.

14. Kersey, P.J., Allen, J.E., Armean, I., Boddu, S., Bolt, B.J., Carvalho-Silva, D., Christensen, M., Davis, P., Falin, L.J., Grabmueller, C., et al. (2016). Ensembl Genomes 2016: more genomes, more complexity. Nucleic Acids Res. *44*, D574–D580.

15. Lek, M., Karczewski, K.J., Minikel, E.V., Samocha, K.E., Banks, E., Fennell, T., O’Donnell-Luria, A.H., Ware, J.S., Hill, A.J., Cummings, B.B., et al. (2016). Analysis of protein-coding genetic variation in 60,706

humans. Nature *536*, 285–291.

16. Thormann, A., Halachev, M., McLaren, W., Moore, D.J., Svinti, V., Campbell, A., Kerr, S.M., Tischkowitz, M., Hunt, S.E., Dunlop, M.G., et al. (2019). Flexible and scalable diagnostic filtering of genomic variants using G2P with Ensembl VEP. Nat. Commun. *10*, 2373.

17. Deciphering Developmental Disorders Study (2017). Prevalence and architecture of de novo mutations in developmental disorders. Nature *542*, 433–438.

18. Thorvaldsdóttir, H., Robinson, J.T., and Mesirov, J.P. (2013). Integrative Genomics Viewer (IGV): high-performance genomics data visualization and exploration. Brief. Bioinform. *14*, 178–192.

19. Firth, H.V., Richards, S.M., Bevan, A.P., Clayton, S., Corpas, M., Rajan, D., Van Vooren, S., Moreau, Y., Pettett, R.M., and Carter, N.P. (2009). DECIPHER: Database of Chromosomal Imbalance and Phenotype in Humans Using Ensembl Resources. Am. J. Hum. Genet. *84*, 524–533.

20. Karczewski, K.J., Francioli, L.C., Tiao, G., Cummings, B.B., Alföldi, J., Wang, Q., Collins, R.L., Laricchia, K.M., Ganna, A., Birnbaum, D.P., et al. (2020). The mutational constraint spectrum quantified from variation in 141,456 humans. Nature *581*, 434–443.

21. Collins, R.L., Brand, H., Karczewski, K.J., Zhao, X., Alföldi, J., Francioli, L.C., Khera, A.V., Lowther, C., Gauthier, L.D., Wang, H., et al. (2020). A structural variation reference for medical and population genetics. Nature *581*, 444–451.

22. Untergasser, A., Cutcutache, I., Koressaar, T., Ye, J., Faircloth, B.C., Remm, M., and Rozen, S.G. (2012). Primer3—new capabilities and interfaces. Nucleic Acids Research *40*, e115–e115.

23. Kaplanis, J., Samocha, K.E., Wiel, L., Zhang, Z., Arvai, K.J., Eberhardt, R.Y., Gallone, G., Lelieveld, S.H., Martin, H.C., McRae, J.F., et al. (2020). Evidence for 28 genetic disorders discovered by combining healthcare and research data. Nature *586*, 757–762.

24. Gardner, E.J., Lam, V.K., Harris, D.N., Chuang, N.T., Scott, E.C., Stephen Pittard, W., Mills, R.E., Devine, S.E., and The 1000 Genomes Project Consortium (2017). The Mobile Element Locator Tool (MELT): population-scale mobile element discovery and biology. Genome Research *27*, 1916–1929.

25. Gardner, E.J., Prigmore, E., Gallone, G., Danecek, P., Samocha, K.E., Handsaker, J., Gerety, S.S., Ironfield, H., Short, P.J., Sifrim, A., et al. (2019). Contribution of retrotransposition to developmental disorders. Nat. Commun. *10*, 4630.

26. Danecek, P., Bonfield, J.K., Liddle, J., Marshall, J., Ohan, V., Pollard, M.O., Whitwham, A., Keane, T., McCarthy, S.A., Davies, R.M., et al. (2021). Twelve years of SAMtools and BCFtools. Gigascience *10*,.
